# Impact of nutritional supplementation during pregnancy on antibody responses to diphtheria-tetanus-pertussis vaccination in infants: A randomised trial in The Gambia

**DOI:** 10.1371/journal.pmed.1002854

**Published:** 2019-08-06

**Authors:** Sandra G. Okala, Momodou K. Darboe, Fatou Sosseh, Bakary Sonko, Tisbeh Faye-Joof, Andrew M. Prentice, Sophie E. Moore

**Affiliations:** 1 Kings’ College London, Department of Women and Children’s Health, St Thomas’ Hospital, London, United Kingdom; 2 MRC Unit The Gambia at the London School of Hygiene and Tropical Medicine, Banjul, The Gambia; London School of Hygiene and Tropical Medicine, UNITED KINGDOM

## Abstract

**Background:**

Exposure to a nutritionally deficient environment during fetal life and early infancy may adversely alter the ontogeny of the immune system and affect an infant’s ability to mount an optimal immune response to vaccination. We examined the effects of maternal nutritional supplementation during pregnancy on infants’ antibody responses to the diphtheria-tetanus-pertussis (DTP) vaccine included in the Expanded Programme on Immunisation (EPI).

**Methods and findings:**

The Early Nutrition and Immune Development (ENID) trial was a randomised, partially blinded trial conducted between April 2010 and February 2015 in the rural West Kiang region of The Gambia, a resource-poor region affected by chronic undernutrition. Pregnant women (<20 weeks’ gestation) with a singleton pregnancy (*n* = 875) were randomised to receive one of four supplements: iron-folic acid (FeFol; standard of care), multiple micronutrient (MMN), protein-energy (PE), or PE + MMN daily from enrolment (mean [SD] 13.7 [3.3] weeks’ gestation) until delivery. Infants were administered the DTP vaccine at 8, 12, and 16 weeks of age according to the Gambian Government protocol. Results for the primary outcome of the trial (infant thymic size) were described previously; here, we report on a secondary outcome, infant antibody response to vaccination. The effects of supplementation on mean DTP antibody titres measured in blood samples collected from infants at 12 weeks (*n* = 710) and 24 weeks (*n* = 662) were analysed with adjustment for confounders including maternal age, compliance to supplement, and infant sex and season. At 12 weeks, following a single dose of the vaccine, compared with FeFol (mean 95% confidence interval [CI]; 0.11 IU/mL, 0.09–0.12), antenatal supplementation with MMN or MMN + PE resulted in 42.4% (95% CI 20.1–64.6; *p* < 0.001) and 29.4% (6.4–52.5; *p* = 0.012) higher mean anti-diphtheria titres, respectively. Mean anti-tetanus titres were higher by 9.0% (5.5–12.5), 7.8% (4.3–11.4), and 7.3% (4.0–10.7) in MMN, PE, and PE + MMN groups (all, *p* < 0.001), respectively, than in the FeFol group (0.55 IU/mL, 0.52–0.58). Mean anti-pertussis titres were not significantly different in the FeFol, MMN, and PE + MNN groups but were all higher than in the PE group (all, *p* < 0.001). At 24 weeks, following all three doses, no significant differences in mean anti-diphtheria titres were detected across the supplement groups. Mean anti-tetanus titres were 3.4% (0.19–6.5; *p* = 0.038) higher in the PE + MMN group than in the FeFol group (3.47 IU/mL, 3.29–3.66). Mean anti-pertussis titres were higher by 9.4% (3.3–15.5; *p* = 0.004) and 15.4% (9.6–21.2; *p* < 0.001) in PE and PE + MMN groups, compared with the FeFol group (74.9 IU/mL, 67.8–82.8). Limitations of the study included the lack of maternal antibody status (breast milk or plasma) or prevaccination antibody measurements in the infants.

**Conclusion:**

According to our results from rural Gambia, maternal supplementation with MMN combined with PE during pregnancy enhanced antibody responses to the DTP vaccine in early infancy. Provision of nutritional supplements to pregnant women in food insecure settings may improve infant immune development and responses to EPI vaccines.

**Trial registration:**

ISRCTN49285450.

## Abstract 2

Sophie Moore and colleagues reveal the enhanced response to the DTP vaccine in newborns in The Gambia following maternal supplementation with micronutrients and protein during pregnancy.

## Author summary

**Why was this study done?:**

- Deficiencies of both macro- and micronutrients are common among pregnant women in low- and middle-income countries (LMICs) and are recognised as a key determinant of poor birth outcomes.
- Nutritional deficiencies during pregnancy may also impact on the longer-term health of the offspring, including immune development and function.
- Our trial was designed to investigate the impact of combined protein-energy (PE) and multiple micronutrient (MMN) supplementation given to women across pregnancy on vaccine responses in young infants.

**What did the researchers do and find?:**

- We performed a randomised, partially blinded trial of nutritional supplementation to pregnant women in rural West Africa, to test whether nutritional supplementation during pregnancy improved antibody response to vaccination in their infants.
- We enrolled 875 pregnant women (mean gestational age at enrolment = 13.7 weeks), who were randomised to receive either iron-folic acid (FeFol; standard of care) alone or combined with MMN, PE, or PE + MMN daily from enrolment until delivery.
- Infants were vaccinated at 8, 12, and 16 weeks of age with the diphtheria-tetanus-pertussis (DTP) vaccine, as recommended by the WHO Expanded Programme on Immunisation (EPI), and their antibody responses were measured at 12 (*n* = 710) and 24 (*n* = 662) weeks of age.
- We observed that supplementation with MMN combined with PE was the most efficient intervention tested, improving mean antibody titres against diphtheria and tetanus at 12 weeks, and tetanus and pertussis titres at 24 weeks.

**What do these findings mean?:**

- Improving the nutritional status of women in areas at high risk of undernutrition has multiple benefits beyond immediate birth outcomes.
- Understanding the mechanisms that link nutritional supplementation during pregnancy to improved vaccine responses in early infancy may lead to the development of novel interventions among vulnerable populations.

## Introduction

The Expanded Programme on Immunisation (EPI), introduced in 1974 by WHO, established a standardised vaccination schedule for all children globally, initially including diphtheria-tetanus-pertussis (DTP), Bacillus Calmette-Guérin (BCG), oral polio, and measles vaccines [1]. This programme has been implemented widely and is estimated to prevent between 2 and 3 million deaths in children annually. However, despite this, there remains a heavy burden of childhood deaths globally, largely from infection-related causes and especially during the first year of life, when the immune system is most vulnerable [2,3]. In tandem, undernutrition during fetal life and early childhood has been estimated to contribute to 45% of all deaths in children globally [4,5]. Through a vicious circle, undernutrition heightens the risk of infections, while infections predispose to undernutrition [5]. In settings such as sub-Saharan Africa, where supplementary nutrition and routine childhood vaccinations are lifesaving [6], novel interventions are required to break this cycle of infection and undernutrition.

The immune system starts to develop early during fetal life so that at birth all components are in place to allow a rapid expansion to complete maturation in infancy and childhood [7]. Nutritional insults during these critical periods may lead to a breakdown in the complex pathways required for its development, with downstream consequences on functionality, including the ability to mount an adequate response to vaccination [8]. A limited number of studies have assessed the impact of direct nutritional supplementation on vaccine responses in children, with findings indicating mixed effects of protein-energy (PE) and micronutrient supplements, including vitamin A, zinc, and iron, across a range of vaccines [9]. Far fewer studies have investigated the effects of maternal nutritional supplementation during pregnancy with a single micronutrient, multiple micronutrients (MMN), or PE on vaccine responses in infancy [10–14]. Overall, the limited data available provide some evidence that nutritional supplementation during fetal development may improve immune response to childhood vaccination [10]. However, the heterogeneity in study design, lack of adjustment for confounders, and limited availability of high-quality data prevents any firm conclusions and supports the urgent need for well-designed trials looking at the effects of antenatal supplementation on vaccine responses in infants.

We report here findings from the Early Nutrition and Immune Development (ENID) trial, a randomised trial conducted in rural Gambia, in an area of widespread and seasonal undernutrition. The ENID trial examined the effects of prenatal and infant nutritional supplementation on infant immune development [15]. The primary outcome of ENID was thymic development during infancy, and antibody response to vaccination was a secondary outcome. We have previously published data showing an effect of infant micronutrient supplementation, but not maternal supplementation, on infant thymic size [16]. Here, we present the effects of maternal supplementation on the secondary outcome, antibody responses to DTP vaccinations in early infancy.

## Methods

### Study design and participants

The ENID trial (ISRCTN49285450) was a partially blinded trial of prenatal and infant nutritional supplementation conducted in the rural West Kiang region of The Gambia between August 2009 (the date of the first participant consented) and February 2015 (the date the last infant born into the trial reached 12 months of age). S1 Fig presents the ENID trial design; full details of the trial are provided in the published trial protocol [15]. The primary outcome measure of the ENID trial was thymic size in infancy (findings reported previously; [16]). Infant antibody response to vaccination (presented here) was a secondary outcome. Other outcomes included measures of cellular markers of immunity in a selected sub-cohort and infant growth to 24 months of age.

Briefly, all women aged 18 to 45 years and registered in the West Kiang Demographic Surveillance System were invited to participate in the study [17], and written informed consent was obtained. Monthly surveillance of all participating women, including a short questionnaire on the date of the last menstrual period, enabled the identification of women with a possible pregnancy, subsequently confirmed by ultrasound examination. All women confirmed to be <20 weeks pregnant were then randomised into the trial, with supplementation commencing the following week until delivery. Women with a gestational age (GA) ≥20 weeks, a multiple pregnancy, severe anaemia (haemoglobin [Hb] <7 g/dL), or confirmed as HIV positive were excluded.

### Interventions

Pregnant women were randomised to one of four intervention arms: (1) Iron-folic acid (FeFol) tablets, representing the usual standard of care as per Gambian Government guidelines; (2) MMN tablets, a combination of 15 micronutrients designed for use during pregnancy as formulated by UNICEF/WHO/UNU (with the exception of FeFol, each tablet contained 2×RDA of each micronutrient [18,19]; (3) protein-energy and iron-folic acid (PE + FeFol) as a lipid-based nutritional supplement (LNS) providing the same level of FeFol as the reference arm, but with the addition of energy, protein, and lipids; (4) protein-energy and multiple micronutrients (PE + MMN) as the same LNS supplement fortified to provide the same level of micronutrients as the MMN arm. The composition of the four supplements is detailed in Table 1. Antenatal supplements were distributed on a weekly basis by community-based field assistants.

**Table 1:** Nutritional composition of the allocated daily intake of pregnancy supplements.

Nutrients	Tablets		LNS	
	FeFol	MMN	PE	MMN + PE
Iron (mg)	60	60	60	60
Folate (μg)	400	400	400	400
Vitamin A (RE μg)		1,600	*2*.*85*	1,600
Vitamin D (IU)		400	*-*	400
Vitamin E (mg)		20	*4*.*2*	20
Vitamin C (mg)		140	*2*.*25*	140
Vitamin B1 (mg)		2.8	*0*.*3*	2.8
Vitamin B2 (mg)		2.8	*0*.*45*	2.8
Niacin (mg)		36	*1*.*35*	36
Vitamin B6 (mg)		2.8	*0*.*15*	2.8
Vitamin B12 (μg)		5.2	*0*.*1*	5.2
Zinc (mg)		30	*3*.*3*	30
Copper (mg)		4	*1*.*05*	4
Selenium (μg)		130	*6*.*15*	130
Iodine (μg)		300	*2*.*6*	300
Energy (kcal)			746	746
Protein (g)			20.8	20.8
Lipids (g)			52.6	52.6

Data in italics represent natural micronutrients content in PE, which is made from the food base ingredients.

Abbreviations: FeFol, iron-folic acid; LNS, lipid-based nutrient supplement; MMN, multiple micronutrients; PE, protein-energy; RE, retinol equivalent.

From six months of age, infants were further randomised to receive either an unfortified LNS paste or the same formulation fortified with MMN. However, for the current analysis, the infant intervention arms will not be considered, as the outcomes included were assessed before the infant supplementation commenced.

### Randomisation

Randomisation was performed in blocks of eight using an automated system reflecting the eight combinations of prenatal and infancy supplements. The antenatal arms of the trial were partly open because it was not possible to blind project staff or study participants to the supplement type (tablet versus LNS); all the investigators, however, were blinded to participant allocation.

### Procedures

Women were invited for a standard antenatal examination at the Medical Research Council (MRC) Keneba clinic at enrolment, and then again at 20 and 30 weeks’ gestation. At each of these visits, maternal anthropometry and ultrasound measures of fetal biometry were taken by a study midwife. Fetal size at the enrolment visit was used to estimate GA. All measurements were performed using standardised and validated equipment and standard operating procedures.

Following delivery, a study midwife visited all women and their newborns for a standard health examination, and infant anthropometric measurements were taken (weight, length, mid-upper arm circumference [MUAC], and head circumference). Infants were subsequently seen at the MRC Keneba field station at 1, 8, 12, 24, and 52 weeks of age, and at home at 16, 20, 32, and 40 weeks for sample collections, health assessments, and anthropometric measurements. The same standard and regularly validated anthropometric equipment was used at each visit (clinic and home visits). At these infant visits, EPI vaccines were also administered, according to the Gambian Government protocol [15]. All study vaccines were acquired from the EPI Department of the Gambian Government and issued by a study nurse, following standard procedures. Briefly, infants were vaccinated at birth (within 72 hours) and at 8, 12, 16, and 40 weeks of age. At birth, they received BCG vaccine, Hepatitis B vaccine (HBV), and oral polio vaccine (OPV). At 8, 12, and 16 weeks of age, they were vaccinated with Penta (diphtheria-Tetanus-pertussis [DTP], HBV, *Haemophilus influenzae* type B [Hib], OPV, and pneumococcal conjugate vaccine [PCV]), and at 40 weeks of age with OPV, measles, and yellow fever vaccines. For the current analysis, only DTP vaccine responses were assessed, using blood samples collected at 12 and 24 weeks of age. DTP responses were measured at 12 weeks, reflecting responses to the first dose of vaccine given at 8 weeks; and at 24 weeks, reflecting antibody responses after up to three doses (at 8, 12, and 16 weeks) of the vaccine. A weekly questionnaire was used to collect data on maternal morbidity (during pregnancy only) and infant morbidity (from birth to 52 weeks) and on infant feeding practices.

### Laboratory analyses

A validated multiple immunoassay based on Luminex xMAP technology was used to measure serum-specific IgG antibody responses directed against the three components of the DTP vaccine: pertussis toxin (Ptx), diphtheria toxoid (Dtxd), and tetanus toxin (Ttx) [20,21]. This assay was chosen as it presents the advantage of specifically measuring Ptx, Dtxd, and Ttx antibodies in a single assay using a small sample volume (5 μL) and small amounts of antigen compared with other ELISA methods. In-house reference standards were used, calibrated against international standards (see below).

Reconstituted freeze-dried Ptx (National Institute for Biological Standards and Control, United Kingdom), Dtxd, and Ttx (Sigma Aldrich, Gillingham, UK) were conjugated to activated carboxylated microspheres (Bio-plex COOH beads, Bio-rad, Watford, UK) using a two-step carbodiimide reaction. The in-house pertussis standard (calibrated against the United States reference pertussis anti-serum human lot 3) was diluted 4-fold in six dilution steps (1:200–1:204,800), whereas the in-house diphtheria-tetanus standard (calibrated against the International Standards NIBSC code Di-10 and NIBSC code TE-3) was diluted 4-fold in eight dilution steps (1:50–819,200). The unknown sera were diluted 1:200 and 1:4,000, whereas the detection antibody, R-Phycoerythrin conjugated goat anti-human IgG (ƴ chain specific) (Jackson ImmunoResearch Laboratories, Westgrove, PA) was diluted 1:200. Results were generated using a Bio-plex 200 system with Bio-plex Manager software (version 4.1.1, Bio-rad, UK). Median fluorescent intensity for Ptx was converted to ELISA Unit (EU)/mL, and for Dtxd and Ttx to International Unit (IU)/mL by interpolation from a five-parameter logistic standard curve. Being responder to the vaccine was defined as presenting an antibody titre >0.1 IU/mL for diphtheria and tetanus, according to international standards (WHO) [20,22]. As for pertussis, an in-house antibody assay was used; an arbitrary threshold was established at >5.0 EU/mL. All antibody assays were performed at the MRC Unit in The Gambia.

### Covariates

Maternal date of birth and age were ascertained from the West Kiang Demographic Surveillance System [17]. Maternal parity was calculated as the number of previous pregnancies, including live births and stillbirths, using data from a questionnaire conducted at enrolment. Enrolled women were asked whether they had attended Arabic and/or English school and for how many years. A binary variable (Yes/No) was generated based on whether women went for at least a year in an Arabic/and or English school, because school attendance was low, with 77.3% (549/710) of women not having attended school. Maternal body mass index (BMI) was computed as weight (kg)/height (m)^2^. Maternal morbidity was calculated as the total number of morbidity episodes during pregnancy divided by the number of weeks enrolled in the study. A compliance score based on the amount of supplement remaining in the jars (empty, half-empty, and full) was generated for LNS products (PE and PE + MMN), and a count of tablets left in the bottle was performed for tablets (MMN and FeFol). For each woman, a compliance percentage was generated by dividing the number of LNS jars or tablets the woman consumed by the number she received and multiplying by 100.

Preterm birth (PTB) was defined as a GA at birth <37 completed weeks and low birth weight (LBW) as birth weight <2,500 g. Exclusively breastfed (EBF) was generated as a binary variable (yes/no) and defined as continuation of exclusive breastfeeding until 12 (or 24) weeks. The infant’s anthropometric measurements at 12 and 24 weeks were converted to z-scores using the WHO Child Growth standards [23]. In the models presented here, weight-for-length-z-scores (WLZs) were used. The infant’s morbidity was generated as total days of reported sickness by the guardian in a weekly questionnaire. As month of vaccination may influence antibody vaccine responses [24], to capture the influence of the month of the first DTP vaccination (at 8 weeks) on antibody responses, the monthly variation was fitted using the first two pairs of the Fourier terms: sin(θ) and cos(θ) and sin(2θ) and cos(2θ) [25]. For the present analyses, the Fourier terms were fitted in pairs and denoted as *F*_1_ = sin(θ) and cos(θ) and *F*_2_ = sin(2θ) and cos(2θ), with θ representing the angle in radians of the date in relation to its position on the annual cycle (on 1 January, θ = 2π/365; on 31 December, θ = 2π). For the infant’s season of birth, a binary variable was generated as rainy = June to October and dry = November to May, to avoid collinearity with the Fourier terms for the month of vaccination.

### Statistical analyses

Statistical analyses were performed using STATA 15·0 (StataCorp LP TX). The study was powered based on thymic index as the primary outcomes [15]. For a power of 80% and a significance level of 5%, we estimated a required total sample size of 847 mother–infant pairs. Statistical significance was set at a two-sided alpha level of *p* < 0.05. Analyses were performed by intention to treat (ITT), including all mother–infant pairs with available antibody data (*N* = 710 at 12 weeks, *N* = 662 at 24 weeks, and *N* = 511 for the analysis of antibody ratios between 12 and 24 weeks). The latter was generated by dividing antibody titres measured at 24 weeks by those measured at 12 weeks for each infant. Descriptive statistics of participants were calculated for each intervention arm. Chi-squared tests for categorical variables or ANOVAs for continuous variables were performed for significant differences in participant characteristics across maternal supplementation groups.

Normality was tested using the Shapiro–Wilk test. Plasma antibody concentrations were skewed; therefore, we used the logarithms (base 10) values of the antibody titres. Linear regression models with robust standard errors adjusted with confounding factors were used to determine the means of antibody titres. Although the use of the robust analysis minimised potential deviations from the assumptions of the multiple linear regression models, further checks were made to the models for linearity and multicollinearity and the residuals were examined to check homoscedasticity and normality. Log-transformed mean antibody titres were compared by Student *t* test and back-transformed from the log scale. Confounding factors were defined a priori based on previous literature and biological plausibility [26,27]. Maternal variables considered as potential effect mediators in the association between supplement status and antibody response to vaccination and included in the linear models were as follows: age and BMI (at baseline), maternal education, supplement group, compliance to supplement, Hb at 30 weeks’ gestation, and maternal morbidity. Infant variables included in the models were GA at delivery, birth season (dry/rainy), month of vaccination, sex, infant size (WLZ), and Hb level at the visit preceding antibody measurements. Mode of feeding and infant morbidity scores were also calculated from birth to the time of antibody assessment (12 or 24 weeks).

Sensitivity analyses were performed to check for any potential bias in the data between infants missing antibody measurements at 12 weeks of age (*n* = 90) or at 24 weeks of age (*n* = 138) compared with those included in the analyses (S1 Table; S2 Table). No significant differences were observed between the characteristics of infants with antibody measurements compared with those missing antibody measurements at either time point. The results of this study, which is part of the ENID trial, are reported in accordance with Consolidated Standards of Reporting Trials (CONSORT) 2010 guidelines.

### Ethical approval

The ENID trial was approved by the Joint Gambian Government/MRC Unit The Gambia ethics committee (SCC1126v2). Written informed consent was obtained from all participants. The trial observed Good Clinical Practice Standards and the current version of the Helsinki Declaration.

## Results

**Fig 1:**
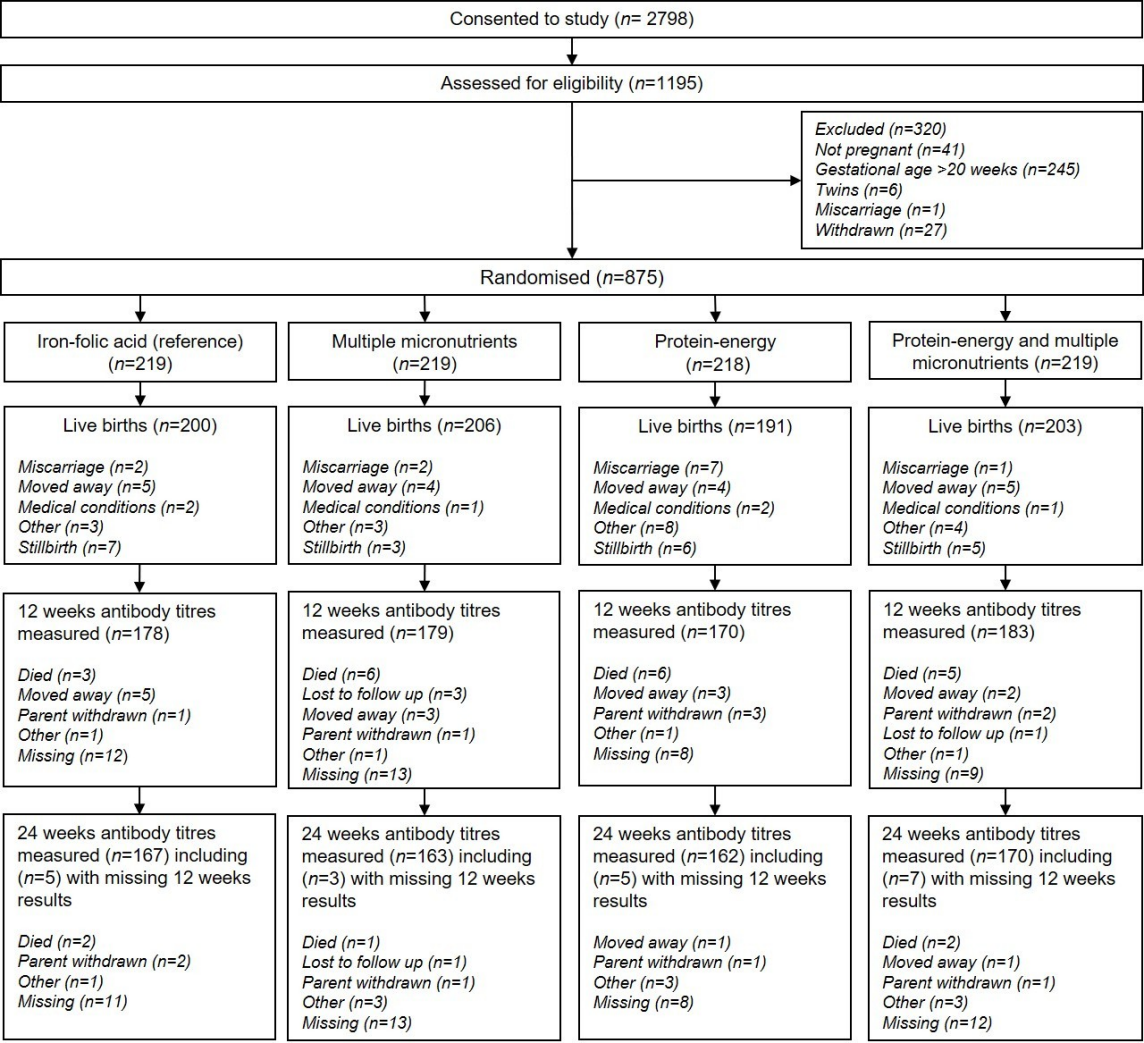
Flow diagram of the trial. CONSORT diagram presenting the flow of participants in the trial. A miscarriage was defined using the WHO definition of the premature loss of a fetus up to 23 weeks of pregnancy. CONSORT, Consolidated Standards of Reporting Trials; FeFol, iron-folic acid; MMN, multiple micronutrients; PE, protein-energy.

A total of 2,798 participants were recruited for monthly surveillance of pregnancy between January 2010 and June 2013, and 1,195 participants were assessed for eligibility (Fig 1). Of these, 875 (73.2%) participants confirmed pregnant with singleton infants and with GA <20 weeks were randomised to enter the antenatal supplementation phase of the trial. Of the 800 live births, 710 (88.8%) infants had DTP antibody measurements at 12 weeks and 662 (82.9%) at 24 weeks and were included in the ITT analyses. There were no differences in participant characteristics between those remaining in the trial and those lost to follow-up, either from baseline to delivery, or from live births to infants included in the current analysis of antibody responses (S1 Table; S2 Table).

Table 2 presents characteristics of the 710 mother-infant by supplement group. At enrolment, mean maternal age was 30.3 (SD 6.8) years, mean GA was 13.7 (3.4) weeks, and 18.9% of women were underweight (<18.5 kg/m^2^). Women were administered antenatal supplements for an average of 26.5 (3.4) weeks. One in five women had received a formal education (22.7%). Mean birth weight was 3.01 (0.40) kg, with 8.9% of the infants being of LBW (<2.50 kg). Mean GA at delivery was 40.2 (1.4) weeks with 5.1% PTB. At 12 weeks of age, the majority of infants (93%) were EBF, compared with half (52%) of infants at 24 weeks. There were no significant differences in maternal or infant characteristics across the supplement groups, except compliance to supplement and maternal Hb levels at 30 weeks’ gestation. Compared with those in the PE or PE + MMN arms (LNS-based supplements), women who received FeFol or MMN (tablets) had a higher compliance to supplementation (93% versus 81%) and a higher mean (SD) Hb level at 30 weeks’ gestation (11.1 [1.2] g/dL versus 10.4 [1.3] g/dL) (both, *p* < 0.001).

**Table 2:** Participant characteristics by maternal supplement group^a^.

	*N*	Maternal supplement groups			
		Tablet supplements		LNS	
		FeFol (*n* = 178)	MMN (*n* = 179)	PE (*n* = 170)	PE + MMN (*n* = 182)
**Maternal variables**	** **				
**Enrolment**	** **				
Age at enrolment (years)	710	30.9 (6.6)	29.5 (6.8)	30.0 (6.5)	30.5 (7.2)
Parity	696	4.5 (2.6)	3.7 (2.6)	4.1 (2.7)	4.3 (2.8)
Formal education, *n* (%)	710	37 (20.8)	47 (26.3)	37 (21.8)	40 (21.9)
GA at enrolment (weeks)	710	13.7 (3.3)	13.9 (3.5)	13.8 (3.3)	13.5 (3.2)
BMI at enrolment (kg/m^2^)	704	21.0 (3.2)	21.1 (3.8)	21.3 (3.6)	21.3 (3.6)
Hb at enrolment (g/dL)	710	11.5 (1.5)	11.4 (1.3)	11.3 (1.4)	11.3 (1.3)
Anaemia at enrolment, *n* (%)^b^	710	60 (33.7)	63 (35.2)	63 (37.1)	72 (39.3)
**30 weeks**	** **				
Hb at 30 weeks’ gestation (g/dL)	658	11.0 (1.2)	11.1 (1.3)	10.3 (1.4)	10.4 (1.3)
Anaemia at 30 weeks, *n* (%)^b^	658	82 (50.0)	69 (42.1)	107 (68.2)	113 (65.3)
**Throughout pregnancy**	** **				
Morbidity events^c^	710	4.4 (3.9)	5.4 (7.5)	5.9 (7.8)	5.4 (6.5)
Compliance to supplement^d^ (% [SD])	710	94.8 (5.0)	92.9 (7.4)	81.2 (15.6)	80.5 (17.4)
**Infant variables**	** **				
**Birth**	** **				
GA at delivery (weeks)	710	40.2 (1.5)	40.3 (1.4)	40.0 (1.4)	40.2 (1.4)
Sex, *n* (%) male	710	90 (50.6)	90 (50.3)	95 (55.9)	91 (49.7)
Dry season birth^e^, *n* (%)	710	106 (59.6)	118 (65.9)	100 (58.8)	119 (65)
Birth weight (kg)	594	2.99 (0.39)	3.00 (0.43)	3.02 (0.4)	3.03 (0.39)
Birth length (cm)	623	49.6 (1.87)	49.53 (2.1)	49.59(1.92)	49.72 (1.89)
**12 weeks**	** **				
Morbidity (days)^f^	710	9.5 (12.6)	9.0 (11.2)	9.3 (11.1)	10.5 (13.5)
Hb (g/dL)	602	10.8 (1.3)	10.7 (1.3)	10.5 (1.5)	10.7 (1.5)
EBF, *n* (%)	710	165 (92.7)	170 (95.0)	162 (95.3)	166 (90.7)
**24 weeks**	** **				
Morbidity (days)	710	23.9 (24.8)	21.8 (23.5)	22.5 (21.8)	23.4 (24.2)
Hb (g/dL)	565	10.5 (1.6)	10.4 (1.4)	10.4 (0.97)	10.6 (1.1)
EBF, *n* (%)	710	102 (57.3)	97 (54.2)	85 (50.0)	85 (46.5)

^a^Values are means (SD) unless stated otherwise.

^b^Anaemia was defined as a Hb level between 7.0 and 10.9 g/dL (WHO).

^c^Number of morbidity episodes between enrolment and delivery.

^d^Compliance percentage was generated by dividing the number of LNS jars or tablets the women consumed by the number she received, and multiplying by 100.

^e^Dry season = November to May.

^f^Number of days of reported sickness between birth and 12 weeks or 24 weeks.

Abbreviations: BMI, body mass index; EBF, exclusively breastfed; FeFol, iron-folic acid (reference); GA, gestational age; Hb, haemoglobin; LNS, lipid-based nutrient supplement, MMN, multiple micronutrients; PE, protein-energy.

Following the first dose of the vaccine (12 weeks), 55.5% of infants presented protective antibody titres for diphtheria, increasing to 96.8% at 24 weeks (after three doses). For tetanus, protective levels were 97.3% after the first dose, increasing to 99.6% at 24 weeks. Finally, for pertussis, this rate increased between 50.1% and 88.2% between doses. For each antigen, no significant differences in the proportion of responders were detected across the supplement groups at 12 or 24 weeks (S3 Table).

**Fig 2:**
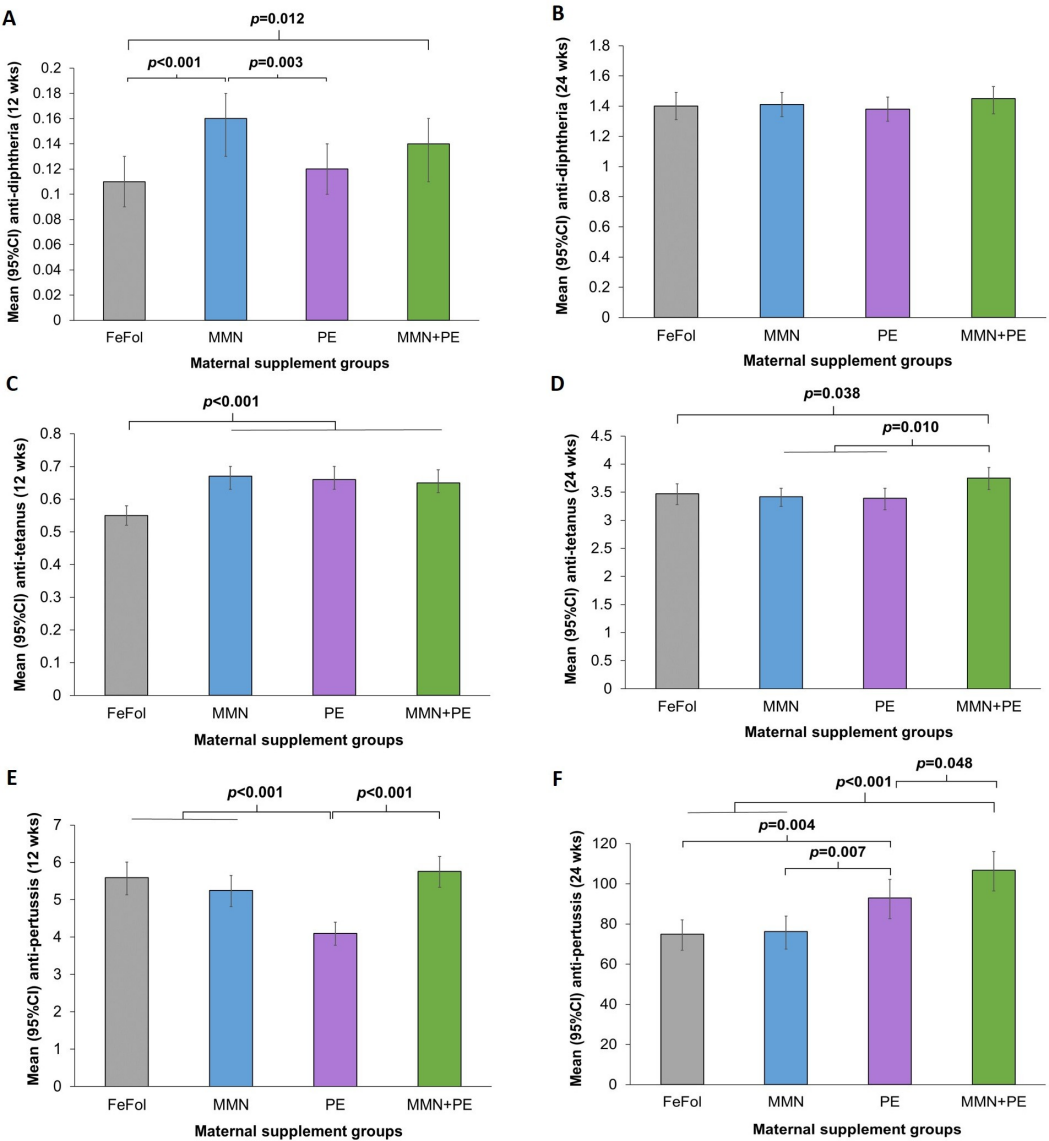
Mean diphtheria, tetanus, and pertussis antibody titres in infants at 12 and 24 weeks of age following the first and third DTP vaccination, by maternal supplement groups. Antibody concentrations were log transformed and, for reporting, mean values and 95% CI were back-transformed from the logarithm scale and expressed in IU/mL for diphtheria and tetanus antibody titres and in EU/mL for pertussis antibody titres. In (A), (C), and (E), means were adjusted with maternal variables: supplement group, compliance to supplement, age, Hb levels at 30 weeks’ gestation, formal education (yes/no), morbidity, and BMI; and with infant variables: GA at delivery, sex, WLZ at first vaccination, Hb levels at 12 weeks, morbidity, EBF (yes/no), birth season (dry/rainy), and Fourier terms of month of the first vaccination. In (B), (D), and (F), means were adjusted with the same factors mentioned above, with changes for infant WLZ third vaccination, Hb levels at 24 weeks, morbidity, and EBF (yes/no) from birth to 24 weeks. Student *t* test was used for comparison between the supplement groups. BMI, body mass index; CI, confidence interval; DTP, diphtheria-tetanus-pertussis; EBF, exclusively breastfed; FeFol, iron-folic acid; GA, gestational age; Hb, haemoglobin; MMN, multiple micronutrients; PE, protein-energy; WLZ, weight-for-length-z-score.

In Fig 2 and Table 3, we present the adjusted mean concentrations of DTP antibodies by supplement groups, along with the effect sizes for the differences between groups. At 12 weeks of infant age, mean anti-diphtheria titres were significantly higher in infants born to mothers who received MMN (0.16 IU/mL, 95% confidence interval [CI] 0.14–0.19) or PE + MMN (0.14 IU/mL, 0.12–0.16) during pregnancy compared with FeFol (0.10 IU/mL, 0.09–0.12). This corresponded to a mean (95% CI) increase in anti-diphtheria concentrations of 42.4% (20.1–64.6) in the MMN group and of 29.4% (6.4–52.5) in the PE + MMN group, compared with FeFol. We also observed that mean (95% CI) anti-diphtheria titres were 33.4% (11.5–55.3) higher in the MMN group compared with the PE group. Compared with the FeFol group, mean (95% CI) anti-tetanus titres were found to be higher by 9.0% (5.5–12.5), 7.8% (4.3–11.4), and 7.3% (4.0–10.7) in the MMN, PE, and PE + MMN groups, respectively. Mean (95% CI) anti-pertussis titres were lower in the PE group (4.1 EU/mL, 3.8–4.4) compared with FeFol (by 13.5%, 8.8–18.2), MMN (by 10.7%, 6.0–15.5), and PE + MMN (by 14.8%, 10.3–19.3) groups.

**Table 3:** Effect size (95% CI) percentage of the comparisons of the mean diphtheria, tetanus, and pertussis antibody titres in infants at 12 and 24 weeks of age across the maternal supplement groups.

Vaccine antigen	Effect size (95% CI) (%) of the comparisons between the supplement groups^a^					
	FeFol	*p*-value^b^	MMN	*p*-value^b^	PE	*p*-value^b^
**12 weeks**	** **	*** ***	** **	*** ***	** **	*** ***
**Diphtheria**						
FeFol	Reference					
MMN	−42.4 (−64.6 to −20.1)	**<0.001**	Reference			
PE	−8.9 (−33.1 to 15.2)	0.467	33.4 (11.5 to 55.3)	**0.003**	Reference	
PE + MMN	−29.4 (−52.5 to −6.4)	**0.012**	12.9 (−8.0 to 33.8)	0.225	−20.5 (−43.3 to 2.3)	0.078
**Tetanus**						
FeFol	Reference					
MMN	−9.0 (−12.5 to −5.5)	**<0.001**	Reference			
PE	−7.8 (−11.4 to −4.3)	**<0.001**	1.2 (−2.3 to 4.7)	0.498	Reference	
PE + MMN	−7.3 (−10.7 to −4.0)	**<0.001**	1.7 (−1.7 to 5.1)	0.321	0.49 (−2.9 to 3.9)	0.779
**Pertussis**						
FeFol	Reference					
MMN	2.8 (−2.0 to 7.6)	0.271	Reference			
PE	13.5 (8.8 to 18.2)	**<0.001**	10.7 (6.0 to 15.5)	**<0.001**	Reference	
PE + MMN	−1.3 (−5.9 to 3.3)	0.903	−4.1 (−8.7 to 0.54)	0.083	−14.8 (−19.3 to −10.3)	**<0.001**
**24 weeks**	** **	** **	** **	** **	** **	** **
**Diphtheria**						
FeFol	Reference					
MMN	−0.35 (−4.0 to 3.3)	0.845	Reference			
PE	0.68 (−3.0 to 4.3)	0.707	1.0 (−2.4 to 4.5)	0.557	Reference	
PE + MMN	−1.7 (−5.5 to 2.0)	0.352	−1.4 (−5.0 to 2.3)	0.454	−2.4 (−6.1 to 1.3)	0.200
**Tetanus**						
FeFol	Reference					
MMN	0.63 (−2.5 to 3.7)	0.688	Reference			
PE	1.0 (−2.3 to 4.3)	0.555	0.36 (−2.8 to 3.6)	0.822	Reference	
PE + MMN	−3.4 (−6.5 to −0.19)	**0.038**	−4.0 (−7.0 to −0.96)	**0.010**	−4.4 (−7.7 to −1.1)	**0.010**
**Pertussis**						
FeFol	Reference					
MMN	−0.74 (−6.9 to 5.4)	0.818	Reference			
PE	−9.4 (−15.5 to −3.3)	**0.004**	−8.7 (−14.9 to −2.4)	**0.007**	Reference	
PE + MMN	−15.4 (−21.2 to −9.6)	**<0.001**	−14.6 (−20.5 to −8.7)	**<0.001**	−6.0 (−11.9 to −0.05)	**0.048**

^a^Effect sizes were determined using the mean difference between two supplement groups from the Student *t* test and were expressed as percentages (%).

^b^The *p*-values were calculated by Student *t* test on the log-transformed antibody concentrations.

Abbreviations: CI, confidence interval; FeFol, iron-folic acid; MMN, multiple micronutrients; PE, protein-energy.

We then compared the mean concentrations of DTP antibodies measured at 24 weeks of infant age between the supplement groups, with effect sizes for each comparison (Fig 2; Table 3). No significant differences in mean anti-diphtheria titres were observed across the groups. In contrast, mean anti-tetanus titres were higher in the PE + MMN group (3.75 IU/mL, 3.56–3.95) compared with FeFol (by 3.4%, 0.19–6.5), MMN (by 4.0%, 0.96–7.0), and PE (by 4.4%, 1.1–7.7) groups. For pertussis, mean antibody titres were higher in the PE (by 9.4%, 3.3–15.5) and PE + MMN groups (by 15.4%, 9.6–21.2), when compared with the FeFol group (74.9 IU/mL, 67.8–82.8). Additionally, mean anti-pertussis titres were higher in the PE group (93.0 EU/mL, 83.7–103.4) compared with the MMN group (by 8.7%, 2.4–14.9) and higher in the PE + MMN group (106.7 IU/mL, 97.4–117.0) compared with both the MMN (by 14.6%, 8.7–20.5) and PE (by 6.0%, 0.05–11.9) groups.

To examine the effects of the supplements on the changes in antibody concentrations between 12 and 24 weeks, we compared the ratios of the adjusted means at 24 and 12 weeks across the supplement groups and calculated the effect sizes for each comparison (Table 4, with unadjusted data presented in S6 Table). The increase in mean anti-diphtheria titres did not differ significantly between the supplement groups. Mean (95% CI) anti-tetanus titres increased more in the FeFol group (6.6-fold, 6.1–7.1) compared with the MMN (5.1-fold, 4.7–5.5), PE (5.2-fold, 4.9–5.6), and PE + MMN groups (5.6-fold, 5.2–6.1). The mean anti-pertussis titre increased by 24-fold (21.8 to 27.5) between 12 to 24 weeks in the PE group, which was a 20.4% (12.9–28), 27.7% (20.5–34.8), and 12.7% (5.9–19.6) higher increase compared with the FeFol, MMN, and PE + MMN groups, respectively. There was also a higher increase in mean anti-pertussis titres in the PE + MMN group compared with FeFol (by 7.7%, 0.45–14.9) and MMN (by 14.9%, 8.1–21.8) groups. Overall, we note that there were few differences between the unadjusted and adjusted models (S4 Table; S5 Table; S6 Table).

**Table 4:** Ratio of the means (95% CI) of diphtheria, tetanus, and pertussis antibody titres between 12 and 24 weeks.

Vaccine antigen	Means ratio^a^ (95% CI)	Effect size (95%CI) (%) of the comparisons between the supplement groups^b^					
		FeFol	*p*-value^c^	MMN	*p*-value^c^	PE	*p*-value^c^
**Diphtheria**							
FeFol	12.5 (9.9 to 16.1)	Reference					
MMN	9.9 (8.4 to 11.7)	11.9 (−0.62 to 24.3)	0.062	Reference			
PE	12.4 (9.0 to 18.0)	0.26 (−12.9 to 13.4)	0.969	−11.6 (−23.6 to 0.45)	0.059	Reference	
PE + MMN	10.7 (8.9 to 13.1)	9.6 (−3.3 to 22.5)	0.143	−2.2 (−14.1 to 9.6)	0.709	9.3 (−3.2 to 21.9)	0.142
**Tetanus**							
FeFol	6.3 (5.8 to 6.8)	Reference					
MMN	5.1 (4.7 to 5.5)	9.0 (4.59 to 13.5)	**<0.001**	Reference			
PE	5.2 (4.9 to 5.6)	8.1 (3.73 to 12.5)	**<0.001**	−0.92 (−5.25 to 3.4)	0.678	Reference	
PE + MMN	5.6 (5.2 to 6.1)	4.6 (0.10 to 9.1)	**0.045**	−4.5 (−8.9 to −0.03)	**0.048**	−3.6 (−7.9 to 0.82)	0.111
**Pertussis**							
FeFol	14.6 (12.8 to 16.8)	Reference					
MMN	12.6 (11.2 to 14.2)	7.2 (−0.22 to 14.7)	0.057	Reference			
PE	24.4 (21.8 to 27.5)	−20.4 (−28.0 to −12.9)	**<0.001**	−27.7 (−34.8 to −20.5)	**<0.001**	Reference	
PE + MMN	18.1 (16.2 to 20.2)	−7.7 (−14.9 to −0.45)	**0.038**	−14.9 (−21.8 to −8.1)	**<0.001**	12.7 (5.9 to 19.6)	**<0.001**

^a^Means were adjusted with maternal variables: supplement group, compliance to supplement, age, Hb levels at 30 weeks’ gestation, formal education (yes/no), morbidity, and BMI; and with infant variables: GA at delivery, sex, WLZ at first DTP vaccination, Hb levels at 12 weeks, morbidity, EBF (yes/no), birth season (dry/rainy), and Fourier terms of month of the first vaccination. Means were back-transformed from the log scale.

^b^Effect sizes were determined using the mean difference between two supplement groups from the Student *t* test and were expressed as percentages (%).

^c^The *p*-values were calculated by Student *t* test on the log-transformed antibody concentrations.

Abbreviations: BMI, body mass index; CI, confidence interval; DTP, diphtheria-tetanus-pertussis; EBF, exclusively breastfed; FeFol, iron folic-acid (reference); GA, gestational age; Hb, haemoglobin; MMN, multiple micronutrient; PE, protein-energy; PE + MMN, protein-energy combined with multiple micronutrients; WLZ, weight-for-length-z-score.

## Discussion

In a food insecure environment, in rural sub-Saharan Africa, infants born to women who received nutritional supplementation during pregnancy containing a combination of MMN and PE had measurably better responses to routine vaccines given in early infancy compared with the standard of care (daily FeFol). To our knowledge, this is the first randomised trial to examine the impact of a comprehensive package of nutritional supplements given during pregnancy on antibody responses to vaccinations within the first 6 months of life. This corroborates previous findings indicating a direct role of maternal nutritional status and supplementation on the infant’s immune development and function.

Although several studies have examined the effects of childhood nutritional status and/or direct supplementation on vaccine responses [9], studies investigating the impact of maternal supplementation during pregnancy on vaccine responses in infants are scarce and have provided mixed results [9]. A recent systematic review identified only nine relevant studies exploring this association, including three observational studies embedded within controlled trials [12,28,29]. A small randomised controlled trial (*N* = 39) conducted in Bangladesh showed that supplementation with zinc (20 mg/day) from the second trimester of pregnancy to 6 months postnatally was weakly associated with antibody responses to HBV postpartum and at 6 months of age (*r* = 0.386; *p* < 0.10) [12]. Conversely, another study in Bangladesh investigating the impact of zinc (30 mg/day) supplementation during pregnancy on immune response to BCG and Hib vaccines in 405 infants found no significant differences in Hib polysaccharide antibody responses between the zinc-supplemented and placebo groups [29]. A study conducted in Birmingham, UK, among 149 Asian infants born to women who participated in a trial of supplementation with PE (10,000–30,000 kcal/trimester) during pregnancy [14] found that at 22 months of age, infants born to supplemented mothers showed an enhanced response to BCG vaccine, assessed by scar formation [28].

The ENID trial was purposefully designed to test whether nutritional repletion of women during pregnancy improved the immune development of their infants. We observed that supplementation with PE combined with MMN was the most efficient intervention tested, improving mean antibody titres against diphtheria and tetanus at 12 weeks, and tetanus and pertussis titres at 24 weeks. This finding supports our original hypothesis that provision of MMN in the format of a large-quantity LNS would be the most efficacious intervention, providing both additional micronutrients and macronutrients to this group of nutritionally vulnerable women. Furthermore, this observation corroborates other data from the literature that show small-quantity LNS (e.g., those with a lower kcal content, but the same quantity of micronutrients) have a mixed impact on birth outcomes [30], whereas high-quantity LNS appear more efficacious [31]. The MMN supplement used in the ENID trial also included a twice-the-RDA micronutrient requirements for pregnant women, with the exception of FeFol, which was set at 60 mg and 400 μg per day, respectively, in line with Gambian Government recommendations. The use of a higher dose of MMN was guided by previous findings from a similar West African population, in which 2×RDA during pregnancy was needed to impact on birth weight [18]. Our findings demonstrate that the provision of PE and MMN to pregnant women confers the greatest benefits to their infants and supports previous observations of a positive impact of combined MMN and early food supplementation on child survival in rural Bangladesh, an area with high rates of maternal and child undernutrition [32].

Our study showed that antenatal supplementation with MMN, compared with the FeFol standard of care, improved responses to diphtheria and tetanus vaccines at 12 weeks but had no measurable benefit on the response to pertussis vaccine at either 12 or 24 weeks. FeFol supplementation is currently recommended by WHO during pregnancy because of its known associations with a reduced risk of adverse pregnancy outcomes, including anaemia, intrauterine growth restriction (IUGR), preeclampsia, PTB, and LBW [33]. However, most pregnant women in developing countries suffer from deficiencies in a range of micronutrients, caused by poor and variety-restricted diets. A recent meta-analysis of individual patient data from 17 randomised trials showed that maternal supplementation with MMN, compared with FeFol alone, improves overall birth outcomes and reduces mortality in female neonates, especially in undernourished and anaemic pregnant women [34]. Our data add to this available evidence and demonstrate that antenatal MMN supplementation promotes immune function in infants born in food insecure settings.

Antenatal supplementation with PE enhanced antibody response to tetanus at 12 weeks and pertussis response at 24 weeks, although no measurable effect was observed with diphtheria. Women getting the LNS (both PE and PE + MMN) received an additional 746 kcal daily. The rationale for the use of this large-quantity LNS was based on the previous findings that protein-energy supplementation during pregnancy significantly improves birth outcomes, including birth weight and perinatal mortality, in this setting [35]. The findings from the current study support the beneficial effect of PE supplementation in pregnancy on immune function. In contrast, however, a negative effect of supplementation with PE was observed for vaccine responses to pertussis at 12 weeks compared with the other groups. This observation is difficult to explain, as it is inconsistent with the other effects observed but may be a consequence of the combined detrimental impact of poor compliance to the LNS products (so in contrast to the FeFol and MMN arms) and lack of MMN (in contrast to the PE + MMN arms). We do not believe the PE group was ‘harmful’; rather, there was some benefit to infants born to women consuming the FeFol as tablets, and so in this case the referent group outperformed the PE group, in which compliance was not as high as anticipated.

We observed for each supplement differences in effect sizes according to the vaccine, with the highest effect sizes measured with diphtheria responses at 12 weeks, suggesting the importance of vaccine-specific factors in modulating the impact of maternal supplements on vaccine responses in infants. A variety of factors may influence antibody responses to vaccination in infants, such as the presence of maternally derived antibodies, parameters inherent to the vaccine itself (e.g., adjuvants, routes of administration, immunogenicity) or to the capacity of the infant to respond to vaccination (e.g., genetics), and other environmental factors (e.g., ongoing infections) [36]. We reported that after the first dose of DTP vaccine, >90% of infants presented protective levels against tetanus compared with approximately 50% of infants against diphtheria and tetanus. Maternal tetanus vaccination during pregnancy is widely implemented in Africa including in The Gambia, and so the presence of maternally derived tetanus antibodies may explain the higher proportion of infants with protective anti-tetanus titres at 12 weeks than with protective anti-pertussis or anti-diphtheria titres at 12 weeks [37]. This may also explain the lower effect size measured with the tetanus response compared with diphtheria and pertussis responses, because there may only be a limited capacity to improve infant responses beyond the benefit conferred by prevaccination levels [37].

Additionally, parameters inherent to the vaccine itself may modulate the effects of the maternal supplements, but as the same adjuvant and route of administration were used in the combined DTP vaccine, the differences in effect sizes observed are likely linked to variations in immunogenicity. Tetanus toxoid vaccine is known to be a stronger immunogen than Dtxd and whole-cell pertussis, especially among immunocompromised individuals such as preterm infants [38]. Therefore, the finding of this study suggests that infant responses to vaccines with poor immunogenicity—such as the recently implemented oral rotavirus vaccine—may be enhanced by improved maternal nutritional status.

We reported significantly higher proportions of infants with protective DTP antibody titres at 24 weeks of age following boosting doses of the vaccine compared with 12 weeks after the priming dose of the vaccine. This increase in antibody titres is expected and related to the prime-boost strategy, which is commonly used to increase the efficacy and immunogenicity of vaccines and augment the numbers of responders after an initial immunisation, and a decline in specific memory B and T lymphocytes. We also observed generally greater effect sizes in relation to the first vaccination and a higher increase in antibody titres among infants, with the lowest responses after the initial immunisation. This suggests that antenatal supplementation may have a greater influence on the responses to the priming dose of a vaccine but few effects on the succeeding doses, which have been designed to allow all the infants, including those with low responses, to recover and reach protective levels.

The main strength of our study is the use of a randomised trial design using a comprehensive nutritional supplementation strategy with robust assessment of antibody responses to DTP, a vaccine which is universally administered in childhood as part of the EPI, and the use of a large representative sample population. DTP antibody titres were measured at 12 and 24 weeks of age, providing an assessment of supplement effects on capacity to mount immune responses after a priming dose and after two boosting doses of the vaccine. Detailed assessment of possible maternal, infant, and environmental effect modifiers enabled a robust evaluation of supplement-mediated effects.

Because the maternal transfer of antibodies through the placenta or breast milk may alter immune responses to vaccination in infancy [39], limitations of this study include the lack of any measures of breast milk antibody levels or prevaccination antibody levels in infants. Furthermore, we recognise that the measured antibody titres in infants may, to a certain extent, be a reflection of maternal antibody concentrations in response to their own vaccination prior to or during pregnancy and, for tetanus at least, the lack of any reliable data on maternal vaccination status reflects a further limitation of the current study. We also acknowledge that antibody measurements may not be an adequate indicator of longer-term immune protection; future studies should therefore incorporate the longitudinal surveillance of morbidity among vaccinated children.

We also note that compliance in the PE and PE + MMN arms of the trial (both given in the form of LNS) was significantly lower than in the FeFol and MMN arms (both given as tablets), and that this was matched by a significantly greater drop in maternal Hb between enrolment and 30 weeks’ gestation in the LNS groups (noting that the daily dose of iron was standard at 60 mg across arms). We have speculated elsewhere that this poorer compliance to this high-quantity LNS may reflect a lower acceptability of these products in this population, rather than a consequence of sharing of the supplement within the community [16]. However, we note that this lower compliance and difference in Hb levels between study arms does not explain the results observed. Finally, the findings from this trial are limited to populations with widespread nutritional deficiencies during pregnancy and with high rates of infection in infants and young children.

In conclusion, we have shown that giving nutritional supplements containing a combination of micronutrients and macronutrients to nutritionally vulnerable pregnant women in rural sub-Saharan Africa improves antibody response to vaccination in early infancy. The effects observed were most marked in response to the priming dose of each component of the DTP vaccine, with less of a marked effect after the full schedule was received. Although the observed effect sizes were modest, even small improvements in antibody response may help an infant to pass the thresholds of unprotective to protective antibody titres. Furthermore, because antibody titres naturally decrease with time following an initial peak after vaccination, higher initial antibody titres may also confer longer protection and improve vaccine effectiveness. Thus, our findings have potential clinical importance indicating that nutritional repletion of pregnant women may help to compensate for immunological vulnerability during the critical period of early infancy. The possibility that the benefits would be greater for vaccines that have lower efficacy in African populations is worthy of further investigation.

## Supporting information

S1 Appendix Trial protocol. (PDF)

S2 Appendix CONSORT checklist. CONSORT, Consolidated Standards of Reporting Trials.
(DOC)

S3 Appendix Ethical application. (DOC)

S4 Appendix Ethical approval. (PDF)

S1 Fig Flow diagram of the trial design. FeFol, iron folic acid; LNS, lipid-based nutritional supplement; MMN, multiple micronutrients; PE, protein-energy.
(TIF)

S1 Table Comparison of characteristics between infants with antibody measurements at 12 weeks of age and those missing data. (DOCX)

S2 Table Comparison of characteristics between infants with antibody measurements at 24 weeks of age and those missing data. (DOCX)

S3 Table Infants respondent, n (%), to each DTP vaccine antigens by supplement group. DTP, diphtheria-tetanus-pertussis.
(DOCX)

S4 Table Comparison of the unadjusted means (95% CI) of diphtheria, tetanus, and pertussis antibody titres at 12 weeks of age, following the first DTP vaccination, by supplement group. CI, confidence interval; DTP, diphtheria-tetanus-pertussis.
(DOCX)

S5 Table Comparison of the unadjusted means (95% CI) of diphtheria, tetanus, and pertussis antibody titres at 24 weeks of age, following three DTP vaccinations, by supplement group. CI, confidence interval; DTP, diphtheria-tetanus-pertussis.
(DOCX)

S6 Table Comparison of the ratio of the unadjusted means (95% CI) of diphtheria, tetanus, and pertussis antibody titres between 12 and 24 weeks of age, following three DTP vaccinations, by supplement group. CI, confidence interval; DTP, diphtheria-tetanus-pertussis.
(DOCX)

## Abbreviations

BCG: Bacillus Calmette-Guérin
BMI: body mass index
CI: confidence interval
CONSORT: Consolidated Standards of Reporting Trials
DTP: diphtheria-tetanus-pertussis
Dtxd: diphtheria toxoid
EBF: exclusively breastfed
ENID: Early Nutrition and Immune Development
EPI: Expanded Programme on Immunisation
FeFol: iron-folic acid
GA: gestational age
Hb: haemoglobin
HBV: Hepatitis B vaccine
Hib: *Haemophilus influenzae* type B
ITT: intention to treat
IUGR: intrauterine growth restriction
LBW: low birth weight
LMIC: low- and middle-income country
LNS: lipid-based nutritional supplement
MMN: multiple micronutrients
MRC: Medical Research Council
MUAC: mid-upper arm circumference
OPV: oral polio vaccine
PCV: pneumococcal conjugate vaccine
PE: protein-energy
PTB: preterm birth
Ptx: pertussis toxin
RDA: recommended dietary allowance
RE: retinol equivalent
Ttx: tetanus toxin
UNU: United Nations University
WLZ: weight-for-length-z-score

## References

[1] WHO | The Expanded Programme on Immunization: World Health Organization; 2013 [updated 2013-12-01 13:51:00] [cited 2018 Nov 5]. Available from: http://www.who.int/immunization/programmes_systems/supply_chain/benefits_of_immunization/en/.

[2] UNICEF W, World Bank, UN-DESA Population Division. WHO | Levels and trends in child mortality report 2018. World Health Organization, 2018 2018-09-21 11:08:31. Report No.

[3] Sanchez-Schmitz G, Levy O. Development of Newborn and Infant Vaccines. Sci Transl Med. 2011;3(90):90ps27 10.1126/scitranslmed.3001880 21734174 PMC4108897

[4] Black RE, Victora CG, Walker SP, Bhutta ZA, Christian P, de Onis M, et al Maternal and child undernutrition and overweight in low-income and middle-income countries. Lancet. 2013;382(9890):427–51. Epub 2013/06/12. 10.1016/S0140-6736(13)60937-X . 23746772

[5] Prendergast AJ. Malnutrition and vaccination in developing countries. Philos Trans R Soc Lond B Biol Sci. 2015;370(1671). 10.1098/rstb.2014.0141 25964453 PMC4527386

[6] Black RE, Allen LH, Bhutta ZA, Caulfield LE, de Onis M, Ezzati M, et al Maternal and child undernutrition: global and regional exposures and health consequences. Lancet. 2008;371(9608):243–60. Epub 2008/01/22. 10.1016/S0140-6736(07)61690-0 . 18207566

[7] Simon AK, Hollander GA, McMichael A. Evolution of the immune system in humans from infancy to old age. Proc Biol Sci. 10.1042/bj2450263 PMC4707740 26702035

[8] Palmer AC. Nutritionally mediated programming of the developing immune system. Adv Nutr. 2011;2(5):377–95. Epub 2012/02/15. 10.3945/an.111.000570 22332080 PMC3183589

[9] Savy M, Edmond K, Fine PE, Hall A, Hennig BJ, Moore SE, et al Landscape analysis of interactions between nutrition and vaccine responses in children. J Nutr. 2009;139(11):2154s–218s. Epub 2009/10/02. 10.3945/jn.109.105312 . 19793845

[10] Obanewa O, Newell ML. Maternal nutritional status during pregnancy and infant immune response to routine childhood vaccinations. Future Virol. 2017;12(9):525–36. 10.2217/fvl-2017-0021 29225661 PMC5716389

[11] Barua P, Chandrasiri UP, Beeson JG, Dewey KG, Maleta K, Ashorn P, et al Effect of nutrient supplementation on the acquisition of humoral immunity to Plasmodium falciparum in young Malawian children. Malar J. 2018;17(1):74 Epub 2018/02/09. 10.1186/s12936-018-2224-6 29415730 PMC5804088

[12] Ahmad SM, Hossain MB, Monirujjaman M, Islam S, Huda MN, Kabir Y, et al Maternal zinc supplementation improves hepatitis B antibody responses in infants but decreases plasma zinc level. Eur J Nutr. 2016;55(5):1823–9. Epub 2015/07/26. 10.1007/s00394-015-0999-6 . 26208687

[13] Zhao X, Pang X, Wang F, Cui F, Wang L, Zhang W. Maternal folic acid supplementation and antibody persistence 5 years after hepatitis B vaccination among infants. Hum Vaccin Immunother. 2018;14(10):2478–84. Epub 2018/06/21. 10.1080/21645515.2018.1482168 29923793 PMC6284482

[14] Viegas OA, Scott PH, Cole TJ, Eaton P, Needham PG, Wharton BA. Dietary protein energy supplementation of pregnant Asian mothers at Sorrento, Birmingham. II: Selective during third trimester only. Br Med J (Clin Res Ed). 1982;285(6342):592–5. 10.1136/bmj.285.6342.592 6819029 PMC1499470

[15] Moore SE, Fulford AJ, Darboe MK, Jobarteh ML, Jarjou LM, Prentice AM. A randomized trial to investigate the effects of pre-natal and infant nutritional supplementation on infant immune development in rural Gambia: the ENID trial: Early Nutrition and Immune Development. BMC Pregnancy Childbirth. 2012;12:107 Epub 2012/10/13. 10.1186/1471-2393-12-107 23057665 PMC3534399

[16] Moore SE, Fulford AJC, Sosseh F, Nshe P, Darboe MK, Prentice AM. Thymic size is increased by infancy, but not pregnancy, nutritional supplementation in rural Gambian children: a randomized clinical trial. BMC Med. 2019;17(1):38 Epub 2019/02/19. 10.1186/s12916-019-1264-2 30773140 PMC6378709

[17] Hennig BJ, Unger SA, Dondeh BL, Hassan J, Hawkesworth S, Jarjou L, et al Cohort Profile: The Kiang West Longitudinal Population Study (KWLPS)-a platform for integrated research and health care provision in rural Gambia. Int J Epidemiol. 2017;46(2):e13 Epub 2015/11/13. 10.1093/ije/dyv206 . 26559544 PMC5837564

[18] Kaestel P, Michaelsen KF, Aaby P, Friis H. Effects of prenatal multimicronutrient supplements on birth weight and perinatal mortality: a randomised, controlled trial in Guinea-Bissau. Eur J Clin Nutr. 2005;59(9):1081–9. Epub 2005/07/15. 10.1038/sj.ejcn.1602215 . 16015266

[19] UNICEF, Organization WH, University UN. Composition of a multi-micronutrient supplement to be used in pilot programmes among pregnant women in developing countries: report of a United Nations Children's Fund (UNICEF), World Health Organization (WHO) and United Nations University workshop. 1999. http://www.who.int/iris/handle/10665/75358.

[20] Colombet I, Saguez C, Sanson-Le Pors MJ, Coudert B, Chatellier G, Espinoza P. Diagnosis of Tetanus Immunization Status: Multicenter Assessment of a Rapid Biological Test. Clin Diagn Lab Immunol. 12 2005 p. 1057–62. 10.1128/CDLI.12.9.1057-1062.2005 16148171 PMC1235798

[21] van Gageldonk PG, van Schaijk FG, van der Klis FR, Berbers GA. Development and validation of a multiplex immunoassay for the simultaneous determination of serum antibodies to Bordetella pertussis, diphtheria and tetanus. J Immunol Methods. 2008;335(1–2):79–89. Epub 2008/04/15. 10.1016/j.jim.2008.02.018 . 18407287

[22] Efstratiou A, Maple CPA, Europe WHOROf. Laboratory diagnosis of diphtheria / by Androulla Efstratiou and P. A. Christopher Maple. 1994. http://www.who.int/iris/handle/10665/108108.

[23] Villar J, Altman DG, Purwar M, Noble JA, Knight HE, Ruyan P, et al The objectives, design and implementation of the INTERGROWTH-21st Project. Bjog. 2013;120 Suppl 2:9–26, v. Epub 2013/05/18. 10.1111/1471-0528.12047 . 23678873

[24] Moore SE, Collinson AC, Fulford AJ, Jalil F, Siegrist CA, Goldblatt D, et al Effect of month of vaccine administration on antibody responses in The Gambia and Pakistan. Trop Med Int Health. 2006;11(10):1529–41. Epub 2006/09/28. 10.1111/j.1365-3156.2006.01700.x . 17002727

[25] Fulford AJ, Rayco-Solon P, Prentice AM. Statistical modelling of the seasonality of preterm delivery and intrauterine growth restriction in rural Gambia. Paediatr Perinat Epidemiol. 2006;20(3):251–9. Epub 2006/04/25. 10.1111/j.1365-3016.2006.00714.x . 16629700

[26] Kampmann B, Jones CE. Factors influencing innate immunity and vaccine responses in infancy. Philos Trans R Soc Lond B Biol Sci. 2015;370(1671). 10.1098/rstb.2014.0148 . 25964459 PMC4527392

[27] de Bruyn G. Cofactors that may influence vaccine responses. Curr Opin HIV AIDS. 2010;5(5):404–8. 10.1097/COH.0b013e32833d1fca 20978381 PMC2978300

[28] Grindulis H, Baynham MI, Scott PH, Thompson RA, Wharton BA. Tuberculin response two years after BCG vaccination at birth. Arch Dis Child. 1984;59(7):614–9. 10.1136/adc.59.7.614 6465929 PMC1628956

[29] Osendarp SJ, Fuchs GJ, van Raaij JM, Mahmud H, Tofail F, Black RE, et al The effect of zinc supplementation during pregnancy on immune response to Hib and BCG vaccines in Bangladesh. J Trop Pediatr. 2006;52(5):316–23. Epub 2006/04/20. 10.1093/tropej/fml012 . 16621858

[30] Ashorn P, Alho L, Ashorn U, Cheung YB, Dewey KG, Harjunmaa U, et al The impact of lipid-based nutrient supplement provision to pregnant women on newborn size in rural Malawi: a randomized controlled trial. Am J Clin Nutr. 2015;101(2):387–97. Epub 2015/02/04. 10.3945/ajcn.114.088617 . 25646337

[31] Huybregts L, Roberfroid D, Lanou H, Menten J, Meda N, Van Camp J, et al Prenatal food supplementation fortified with multiple micronutrients increases birth length: a randomized controlled trial in rural Burkina Faso. Am J Clin Nutr. 2009;90(6):1593–600. Epub 2009/10/09. 10.3945/ajcn.2009.28253 . 19812173

[32] Persson LA, Arifeen S, Ekstrom EC, Rasmussen KM, Frongillo EA, Yunus M. Effects of prenatal micronutrient and early food supplementation on maternal hemoglobin, birth weight, and infant mortality among children in Bangladesh: the MINIMat randomized trial. Jama. 2012;307(19):2050–9. Epub 2012/06/06. 10.1001/jama.2012.4061 . 22665104

[33] WHO | WHO recommendations on antenatal care for a positive pregnancy experience. World Health Organization, 2018 2018-12-05 14:15:38. Report No. 28079998

[34] Smith ER, Shankar AH, Wu LS, Aboud S, Adu-Afarwuah S, Ali H, et al Modifiers of the effect of maternal multiple micronutrient supplementation on stillbirth, birth outcomes, and infant mortality: a meta-analysis of individual patient data from 17 randomised trials in low-income and middle-income countries. Lancet Glob Health. 2017;5(11):e1090–e100. Epub 2017/10/14. 10.1016/S2214-109X(17)30371-6 . 29025632

[35] Ceesay SM, Prentice AM, Cole TJ, Foord F, Weaver LT, Poskitt EM, et al Effects on birth weight and perinatal mortality of maternal dietary supplements in rural Gambia: 5 year randomised controlled trial. Bmj. 1997;315(7111):786–90. Epub 1997/11/05. 10.1136/bmj.315.7111.786 9345173 PMC2127544

[36] Saso A, Kampmann B. Vaccine responses in newborns. Semin Immunopathol. 2017;39(6):627–42. 10.1007/s00281-017-0654-9 . 29124321 PMC5711983

[37] Voysey M, Kelly DF, Fanshawe TR, Sadarangani M, O’Brien KL, Perera R, et al The Influence of Maternally Derived Antibody and Infant Age at Vaccination on Infant Vaccine Responses: An Individual Participant Meta-analysis. JAMA Pediatr. 2017;171(7):637–46. 10.1001/jamapediatrics.2017.0638 . 28505244 PMC5710349

[38] Baxter D. Vaccine responsiveness in premature infants. Hum Vaccin. 2010;6(6):506–11. Epub 2010/06/04. 10.4161/hv.6.6.12083 . 20519938

[39] Edwards KM. Maternal antibodies and infant immune responses to vaccines. Vaccine. 2015;33(47):6469–72. Epub 2015/08/11. 10.1016/j.vaccine.2015.07.085 . 26256526

