# Supplementary material for: Impact of nutritional supplementation during pregnancy on antibody responses to diphtheria-tetanus-pertussis vaccination in infants: A randomised trial in The Gambia
Source: PLoS Med. 2019 Aug 6;16(8):e1002854. doi: 10.1371/journal.pmed.1002854 (PMC6684039; doi:10.1371/journal.pmed.1002854)
Supplement: S1 Appendix — (PDF) [file pmed.1002854.s001.pdf]

Moore et al. BMC Pregnancy and Childbirth 2012, 12:107
http://www.biomedcentral.com/1471-2393/12/107

BMC Pregnancy & Childbirth

STUDY PROTOCOL Open Access

# A randomized trial to investigate the effects of pre-natal and infant nutritional supplementation on infant immune development in rural Gambia: the ENID trial: Early Nutrition and Immune Development

Sophie E Moore[1,2*], Anthony JC Fulford[1,2], Momodou K Darboe[1], Modou Lamin Jobarteh[1], Landing M Jarjou[1] and Andrew M Prentice[1,2]

## Abstract

**Background:** Recent observational research indicates that immune development may be programmed by nutritional exposures early in life. Such findings require replication from trials specifically designed to assess the impact of nutritional intervention during pregnancy on infant immune development. The current trial seeks to establish: (a) which combination of protein-energy (PE) and multiple-micronutrient (MMN) supplements would be most effective; and (b) the most critical periods for intervention in pregnancy and infancy, for optimal immune development in infancy.

**Methods/Design:** The ENID Trial is a 2 x 2 x 2 factorial randomized, partially blind trial to assess whether nutritional supplementation to pregnant women (from < 20 weeks gestation to term) and their infants (from 6 to 12 months of age) can enhance infant immune development. Eligible pregnant women from the West Kiang region of The Gambia (pregnancy dated by ultrasound examination) are randomized on entry to 4 intervention groups (Iron-folate (FeFol = standard care), multiple micronutrients (MMN), protein-energy (PE), PE + MMN). Women are visited at home weekly for supplement administration and morbidity assessment and seen at MRC Keneba at 20 and 30 weeks gestation for a detailed antenatal examination, including ultrasound. At delivery, cord blood and placental samples are collected, with detailed infant anthropometry collected within 72 hours. Infants are visited weekly thereafter for a morbidity questionnaire. From 6 to 12 months of age, infants are further randomized to a lipid-based nutritional supplement, with or without additional MMN. The primary outcome measures of this study are thymic development during infancy, and antibody response to vaccination. Measures of cellular markers of immunity will be made in a selected sub-cohort. Subsidiary studies to the main trial will additionally assess the impact of supplementation on infant growth and development to 24 months of age.

**Discussion:** The proposed trial is designed to test whether nutritional repletion can enhance early immune development and, if so, to help determine the most efficacious form of nutritional support. Where there is evidence of benefit from a specific intervention/combination of interventions, future research should focus on refining the supplements to achieve the optimal, most cost-effective balance of interventions for improved health outcomes.

**Trial registration:** ISRCTN49285450

* Correspondence: smoore@mrc.gm
[1]MRC Keneba, MRC Unit The Gambia, PO Box 273, Banjul, The Gambia
[2]MRC International Nutrition Group, London School of Hygiene and Tropical Medicine, Keppel Street, London WC1E 7HT, UK

BioMed Central

© 2012 Moore et al.; licensee BioMed Central Ltd. This is an Open Access article distributed under the terms of the Creative Commons Attribution License (http://creativecommons.org/licenses/by/2.0), which permits unrestricted use, distribution, and reproduction in any medium, provided the original work is properly cited.

## Background

### General evidence for nutritional programming of immunity

A substantive and continually expanding body of observational evidence supports the thesis that nutritional exposures during pregnancy and/or early infancy may programme developing organ systems and homeostatic pathways, with later consequences for disease outcomes – the 'Developmental Origins of Health and Disease' hypothesis (DOHaD) [1]. Whilst most research to date within this field has focused on programming of chronic degenerative disease, evidence also exists to suggest that the developing immune system may be particularly vulnerable to early-life insults from nutritional inadequacies [2]. Such defects would result in later life deficiencies in immuno-competence and susceptibility to morbidity and mortality from infectious disease.

In rural Gambia, many aspects of health and behaviour are defined by the distinct seasonal pattern, with a long dry season from November to June followed by a period of intense rainfall lasting from July to October. Pregnant women are especially susceptible to these effects, with seasonal patterns observed in pregnancy weight gains [3], length of gestation [4] and birth weight [5]. We have previously observed that, in this community, birth season predicts infection-related mortality, providing evidence that seasonal factors in early life may programme immune development [6,7]. Data from prospective birth cohort studies in The Gambia [8] and Bangladesh [9] demonstrate environmental effects on thymic size and, in The Gambia, on markers of cellular immunity [10,11]. Cohort studies in adolescents and adults from Philippines [12] and Pakistan [13,14] respectively link early life nutritional exposures to possible long term defects in functional immunity, as assessed by antibody response to vaccination.

To date, however, the majority of the published research in this area from our group and others has focused on observational studies designed to unravel the biological mechanisms underpinning early-life programming of immune function. Rather than continue with such studies within this field, we have initiated a proof-of-principle intervention trial employing comprehensive multiple micronutrient (MMN) and protein-energy (PE) supplements to both mother during pregnancy and her infant from six-months of age in The Gambia. The recent development of affordable lipid-based nutritional supplements (LNS) allows a direct comparison between different routes of MMN administration. To date, LNS have widely been used successfully in the treatment of malnutrition and a wider research agenda is currently looking at the use of LNS for the prevention of malnutrition. LNS outperforms other forms of MMN supplementation in terms of growth; in a recent trial in Ghana, 3 types of micronutrient supplements for home fortification of complementary foods were compared [15]. The most effective supplement was found to be an energy-dense peanut-based fortified spread 'Nutributter' [16]. Additional advantages of LNS favouring translational applications include local production, long shelf life and safety.

The aim of the current trial is to test the hypothesis that early-life immunocompetence can be enhanced by a 'life-course' approach involving LNS to achieve nutritional repletion in late gestation and infancy, with consequent benefits in reducing vaccine failures and morbidity, and enhancing growth. The proposed design will specifically be able to test three exposures: (i) main effects due to each supplement; (ii) comparisons of main effects of different supplements; and (iii) effect modification of one supplement by another.

## Methods/Design

### Study design

The current trial has been designed to specifically investigate the effects of pre-natal and infant nutritional supplementation on infant immune development in rural Gambia, and has been given the acronym ENID: Early Nutrition and Immune Development. The ENID trial is a $2 \times 2 \times 2$ factorial randomized partially blind trial of nutritional supplementation to pregnant women and their infants in the West Kiang region of The Gambia. Within the trial design, women are randomized to supplementation from when they book for antenatal care (< 20 weeks gestation) until delivery and then their infants are further randomized from 6 to 12 months of age. The primary outcome measurements focus on infant immune development. Recruitment into the ENID trial started in early 2010, with the first infant born in August that year. It is anticipated that antenatal recruitment will be completed in December 2012, with the final infant reaching 12 months of age by approximately August 2014.

### Setting and participants

The ENID Trial is based in the West Kiang region of The Gambia, a rural subsistence farming community of savannah and farmland, a roughly 750 $km^2$ rectangular tract of farmland bounded on 3 sides by the River Gambia and its tributaries. The trial is based in all 36 villages currently registered within the West Kiang Demographic Surveillance System (DSS; total resident population approximately 15,000). Following approval from the local community, all women of reproductive age (18 to 45 years) are invited to participate, and informed consent obtained. For the purpose of informed consent, a trained field worker explains the full details of the study to the subject, covering all aspects of the study as laid out in an information sheet. Illiterate

subjects additionally have the full information sheet read to them; literate subjects are given time to read the information sheet in their own time. Any questions that arise are then answered by the field worker, or referred to the principal investigator for clarification. Subjects are also given the possibility to speak to one of the study investigators (PI, study midwife, study clinician) if they wish. If the subject agrees to participate, written consent is obtained, through either a signature or thumb print. Women who are (i) currently pregnant (beyond 20 weeks on ultrasound assessment), (ii) currently enrolled in another MRC study, (iii) severely anaemic at booking (haemoglobin (Hb) less than 7 g/dL), or (iv) report the onset of menopause are excluded from entry into the trial.

Each month from enrolment participating women are visited by a member of the study team with a short questionnaire on the date of their last menstrual period (LMP). When a menses has been missed, a urine sample is collected for pregnancy testing using hCG tests (QuickVue™ One-Step hCG Urine Test, bioMérieux, UK). Women with a positive test are then invited to MRC Keneba for an ultrasound examination. Women confirmed as being between 10–20 weeks pregnant by ultrasound (using a Siemens ACUSON Antares Ultrasound Imaging System (Siemens Medical Solutions USA, Inc., California, USA) with a CH6-2 (5.71 MHz) transducer), are randomized into the trial, with supplementation commencing the following week.

The ENID Trial aims to have complete data for 800 mother-infant pairs. To account for losses to follow up (including those from withdrawals, out-migrations, pregnancy losses, infant deaths), ethical approval was obtained to recruit up to 1000 pregnant women, to allow for a maximum attrition rate of 20%. We anticipate, however, that fewer than 1000 women will need to be recruited, as the West Kiang population is highly motivated for participation in research projects.

The trial is approved by the joint Gambia Government / MRC The Gambia Ethics Committee (Project number SCC1126v2). The trial is monitored by an Independent Trial Monitor (ITM; Dr Karen Edmond, London School of Hygiene and Tropical Medicine) and a Data Safety Monitor (DSM; Dr Kalifa Bojang, Royal Victoria Teaching Hospital, Banjul, and MRC Unit, The Gambia). The primary role of the ITM is to monitor adherence to the trial protocol and to supervise the progress of the trial toward its objectives. The DSM will conduct interim analyses of the safety data, by randomization group, pertaining to the occurrence of Adverse Events (AE); (i) following recruitment of one half of antenatal cases, (ii) on completion of antenatal recruitment, and (iii) once half the infants have reached the final point in the trial, at 12 months of age. In addition, reports on Serious Adverse Events (SAE) are submitted to the DSM in real time. The trial will be stopped in the case of (A) any unexpected SAE (such as a severe/fatal allergic reaction to any one of the trial supplements) or (B) a consistent pattern of SAEs and/or AEs within one or more arms of the trial, and following the direction of both the DSM and ITM. No interim analyses on the outcome data are planned.

## Intervention - pregnancy

At booking eligible women are randomized to one of four intervention arms: 1.) Iron-folate (FeFol), representing the usual standard of care during pregnancy, as per Gambian Government guidelines; 2.) Multiple micronutrients (MMN), a combination of 15 micronutrients, specifically designed for use during pregnancy, and as formulated by UNICEF/WHO/UNU. A single tablet provides the Recommended Dietary Allowance (RDA) for each micronutrient. However, in view of evidence to suggest that in this region a daily supplement of twice the RDA is more effective with regard to birth outcomes [17], women in this arm of the trial are supplemented at 2 × RDA (but with the same level of iron and folate to the FeFol arm). Both the FeFol and MMN supplements are formulated as tablets and manufactured by Scanpharm, Denmark (www.scanpharm.dk). 3.) Protein-energy and iron-folate (PE + FeFol), a lipid based nutritional supplement (LNS) providing the same level of iron and folate to the FeFol only arm, but with the addition of energy, protein and lipids. 4.) Protein-energy and multiple micronutrients (PE + MMN), a micronutrient fortified LNS supplement providing the same level of micronutrients to the MMN arm (including FeFol), in addition to the energy and protein and lipid content. The two LNS products are manufactured by Valid Nutrition, Nairobi, Kenya (www.validinternational.org). The composition of the four pre-natal supplement groups is as detailed in Table 1.

## Randomization and allocation

Randomization into the trial is in blocks of 8, using an automated system, with the 8 groups reflecting the 8 combinations of prenatal and infancy supplements (Figure 1). Once randomized, the code allocated to each mother will remain the same for her infant. Allocation of each supplement combination to a number between 1 and 8 was performed by Dr Mathilde Savy (IRD, France), with this information passed directly to the supplement manufacturers. Each box of supplement is then distinguished by a number between 1 and 8. An additional hard copy of the code assignment is held in the safe in Keneba, accessible by the field station senior administrator and only at the request of the trial monitors. The antenatal arm of the trial is partly open, since it is not be possible to

**Table 1 Nutritional composition of daily intake of pregnancy supplements**

| | Tablets | | LNS | |
|---|---|---|---|---|
| | FeFol | MMN | PE + FeFol | PE + MMN |
| Iron (mg) | 60 | 60 | 60 | 60 |
| Folate (µg) | 400 | 400 | 400 | 400 |
| Vitamin A (RE µg) | | 1600 | *2.85* | 1600 |
| Vitamin D (IU) | | 400 | - | 400 |
| Vitamin E (mg) | | 20 | *4.2* | 20 |
| Vitamin C (mg) | | 140 | *2.25* | 140 |
| Vitamin B1 (mg) | | 2.8 | *0.3* | 2.8 |
| Vitamin B2 (mg) | | 2.8 | *0.45* | 2.8 |
| Niacin (mg) | | 36 | *1.35* | 36 |
| Vitamin B6 (mg) | | 2.8 | *0.15* | 2.8 |
| Vitamin B12 (µg) | | 5.2 | *0.1* | 5.2 |
| Zinc (mg) | | 30 | *3.3* | 30 |
| Copper (mg) | | 4 | *1.05* | 4 |
| Selenium (µg) | | 130 | *6.15* | 130 |
| Iodine (µg) | | 300 | *2.6* | 300 |
| Energy (kcal) | | | 746 | 746 |
| Protein (g) | | | 20.8 | 20.8 |
| Lipids (g) | | | 52.6 | 52.6 |

Data in italics represent MMN content from base ingredients.

blind the field assistants or the women to the supplement type (tablet *vs*. LNS); all other investigators however will not know to which group the women belong.

Using the automated allocation system, a member of the data office at MRC Keneba, independent to the trial analysis, allocates mother-infant pairs to their supplement codes and then generates printed labels for the supplement pots (including subject ID, name, and date of supplement period). Four members of the MRC Keneba field staff working on a different study then label the supplements using lists, by supplement allocation number (e.g. Group 1, women a, b, c etc.) provided by the data office. Supplements are then administered to the women on a weekly basis by field assistants posted to the community. Compliance with the supplement is assessed through the collection of all unused supplements at the end of each week. At these weekly visits, a record of maternal morbidity is also collected. Random spot checks to assess used and unused supplements are additionally performed on 10% of women each week. Details of any other maternal morbidity requiring medical care will be captured electronically through the Keneba clinic or, for cases seen at either of the other two Government Health Centres in West Kiang (Karantaba and Kwinella), through a 'Maternal Morbidity' Form held by health personnel.

### Scheduled pregnancy and delivery measurements and sample collection details

At 20 and 30 weeks gestation, women attend the MRC Keneba clinic for a detailed antenatal assessment. At each visit, the following assessments are made: Standard antenatal check: including blood pressure; haemoglobin; urine sample. Women are also offered voluntary counseling and testing (VCT) for HIV, as is routine practice in West Kiang. Maternal anthropometry: weight; standing height; sitting height; lower leg length; waist and hip circumference and mid-upper-arm circumference (MUAC). All measurements are made using standard, regularly validated equipment and following the relevant SOP. Venous blood sample: haematology and plasma micronutrient

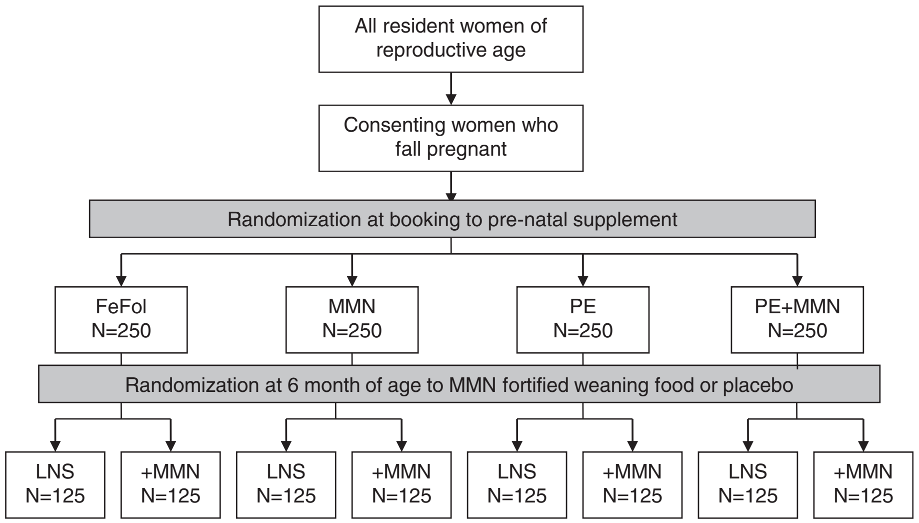


**Figure 1** Trial design.

status, plus samples of serum, plasma and DNA are biobanked for future analyses. Fetal ultrasound: Fetal biometry by ultrasound (Siemens ACUSON, as above), including bi-parietal diameter, occipital frontal diameter, head circumference, abdominal circumference and femur and tibia length.

In this community, the majority of women choose to deliver in their homes with the support of a Traditional Birth Attendant (TBA). Through a network of posted field workers, we attempt to attend all deliveries occurring in West Kiang. Following delivery a sample of cord blood (15 mL) is collected and the placenta transported on ice to the MRC Keneba laboratory for processing. On arrival, cord blood samples are processed and placental material is collected for the assessment of placental micronutrient transport proteins.

Within 72 hours of delivery, all women and their newborn infants are visited by one of the study midwives for a ‘baby check,’ which includes infant anthropometry (weight, length, MUAC, head circumference), gestational age assessment by Dubowitz score [18] and a general health check. Neonates assessed as unwell are referred to the MRC Keneba clinic for assessment by the study clinician/resident paediatrician.

### Intervention - infancy

In The Gambia, exclusive breastfeeding to six months of age is recommended and the National Nutrition Agency (NaNA) actively promotes this practice to the population. Community-based peer counselling in exclusive breastfeeding has proven efficacious in improving both the rate and duration of exclusive breastfeeding [19,20]. Within the ENID Trial, we will work with NaNA to train community health nurses to promote exclusive breastfeeding for all participating women. The purpose of this element of the study is to ensure that all women are provided with the knowledge to provide optimal nutrition to their infants during the period from birth to 6 months of age.

Between birth and six months of age, infants are visited weekly for the collection of morbidity data by questionnaire. From six months of age, infants commence supplementation with either an unfortified LNS paste or the same LNS formulation fortified with multiple micronutrients. The nutritional composition of both products is as detailed in Table 2. This arm of the trial is double blind, with infants receiving identically packaged formulations prepared in individually labeled plastic containers. Supplements are distributed to mothers on a weekly basis, and the mother is asked to administer the supplement/placebo on a daily basis by mixing 20 g of the LNS product with the infant’s normal weaning food. Identical spoons are provided to mothers, to encourage that a uniform amount is given to each infant, per day. Mothers are asked to give the study infant the full dose, and compliance is assessed through random spot checks and by a questionnaire administered on a weekly basis. At these weekly visits, a morbidity questionnaire is also completed for each infant.

**Table 2 Nutritional composition of 20 g of weaning food fortificant**

| Nutrient | PE | PE + MMN |
|---|---|---|
| β-Carotene (μg RE) | *1.84* | 400 |
| Vitamin C (mg) | *1.88* | 30 |
| Folic acid (μg) | *13.1* | 80 |
| Thiamine (mg) | *0.06* | 0.3 |
| Riboflavin (mg) | *0.04* | 0.4 |
| Vitamin B3 (mg) | *0.32* | 4 |
| Pantothenic acid (mg) | *0.08* | 1.8 |
| Vitamin B6 (mg) | *0.02* | 0.3 |
| Vitamin B12 (μg) | *0.06* | 0.5 |
| Vitamin D (μg) | *0.34* | 5 |
| Vitamin E (mg) | *0.12* | 2.7 |
| Vitamin K (μg) | *1.40* | 10 |
| Iron (mg) | *0.46* | 9 |
| Zinc (mg) | *0.24* | 4 |
| Calcium (mg) | *33.1* | 100 |
| Potassium (mg) | *91.76* | 152 |
| Copper (mg) | *0.02* | 0.2 |
| Selenium (μg) | *1.44* | 10 |
| Iodine (μg) | *1.40* | 90 |
| Phosphorus (mg) | *42.56* | 82 |
| Magnesium (mg) | *14.56* | 16 |
| Manganese (mg) | *0.08* | 0.08 |
| Total energy (kcal) | 108 | 108 |
| Linoleic acid (g) | 1.29 | 1.29 |
| Linolenic acid (g) | 0.29 | 0.29 |

Data in italics represent MMN content from base ingredients.

### Scheduled infancy measurements and sample collection details

Following delivery, infants are seen at the MRC Keneba field station when they are 1, 8, 12, 24 and 52 weeks of age, with additional home visits at 16, 20, 32 and 40 weeks of age. Table 3 details the data and sample collection schedule for each visit. At all time points, detailed measures of infant anthropometry are taken using standard protocols with regularly validated equipment. Measurements include weight, length, knee-heel length, head circumference, abdominal circumference, chest circumference, thigh circumference, MUAC, and triceps, biceps, subscapular, suprailiac and thigh skinfold thickness. At all visits in Keneba, a 5 mL sample of breast milk is also collected from each breast, for

**Table 3 Data and sample collection schedule during infancy**

| Measure | Infant age (weeks) | | | | | | | | | | | | | |
|---|---|---|---|---|---|---|---|---|---|---|---|---|---|---|
| | 1 | 4 | 8 | 12 | 16 | 20 | 24 | 28 | 32 | 36 | 40 | 44 | 48 | 52 |
| Thymic Index | + | | + | | | | + | | | | | | | + |
| EPI Vaccination | + | | + | + | + | | | | | + | | | | |
| Venous Bleed | | | | + | | | + | | | | | | | + |
| Anthropometry | + | | + | + | + | + | + | | + | + | | | | + |
| Breast milk | + | | + | + | | | + | | | | | | | + |

the assessment of breast milk micronutrient levels and residual samples frozen at −80°C for long term storage. Milestones of Gross Motor Development are assessed monthly, from when the child is six months of age [21]. At 12 months of age, parental anthropometry is taken and a socio-economic questionnaire completed.

Study personnel administer EPI vaccines to all infants, as per Gambian government protocol (Table 4). All study vaccines are acquired direct from the EPI Department of the Gambia Government, and the cold-chain managed thereafter by the study team. At 12, 24 and 52 weeks of age, a venous blood sample is collected from infants for the assessment of antibody response to vaccination, micronutrient status, with residual aliquots biobanked for further biomarker measurements. Cord blood and samples from 12 and 24 week bleeds will be used to assess anti-diphtheria, tetanus toxoid, Haemophilus B Influenzae, OPV and Hepatitis B antibody titres will be measured. In cord, 24 week and 52 week samples, measles antibody levels will be assessed. DNA samples for both mother and infant are collected and held within the Keneba Biobank.

Thymus size is assessed sonographically at 1, 8, 24 and 52 weeks using a validated method in which the transverse diameter of the thymus and the saggital area of its largest lobe are multiplied to give a volume-related thymic index (TI) [22]. The human thymus is a primary lymphoid organ essential for the establishment of a normal peripheral T-lymphocyte immune system. Whilst little data exists to support TI as a robust measure of immunocompetence, TI has been shown to correlate with thymus weight at autopsy and has been used previously by our group both in Keneba and in Bangladesh to show that the human thymus is sensitive to environmental influences during infancy [8]. Thymus size will be measured using a Siemens Acuson ultrasound unit together with a P10-4 Transducer (Siemens, UK). To further validate the significance of TI and to understand the cellular basis of any observed alterations in antibody response, we will measure cellular markers of immunity in a sub-sample of the cohort (one full calendar year of births), using samples of infant blood collected at 12, 24 and 52 weeks of age.

**Table 4 EPI vaccine schedule, birth to 12 months – The Gambia**

| Age (wk) | Vaccine |
|---|---|
| **0 (within 72 hours of birth)** | **BCG, HBV, OPV** |
| 8 | Penta, OPV, Pneumo |
| 12 | Penta, OPV, Pneumo |
| 16 | Penta, OPV, Pneumo |
| 40 | Measles, Yellow Fever, OPV |

*BCG* – Bacillus Calmette-Guérin; *HBV* – Hepatitis B Vaccine; *OPV* – Oral Polio Vaccine; *Penta* – Combined DPT (diphtheria, pertussis, tetanus), *HBV* and Haemophilus influenzae Type B (Hib).

## Study outcomes

The primary outcome measure for this study is TI. As with previous datasets on thymic development [9] we will assess the impact of the interventions on TI at the different time points (week 1, 8, 24 & 52) separately, and on the time points pooled. These separate analyses are deemed necessary, since the different time points reflect different exposures (week 1 measurements reflecting prenatal supplementation only, week 52 reflecting combined pre- and post-natal interventions). Secondary outcomes include (i) antibody response to vaccination and (ii) detailed cellular immunology (on a subset of infants only).

## Analytical plan

The ENID Trial aims to have complete data on a total of 800 mother-infant pairs. There are three types of questions that the proposed study will address:

(i) Main effects due to each supplement (i.e. supplement *vs.* placebo (usual care; FeFol only)).
(ii) Comparison of main effects of different supplements (i.e. supplement A *vs.* supplement B).
(iii) Effect modification of one supplement by another.

In each case we consider analysis of both (a) the full data set (i.e. all combinations of treatment A, B & C; subgroups 1–8 in Table 5) and and (b) also (in order to avoid possible interference from interactions) in the subset of subjects who did not receive any supplements

**Table 5 Treatment combinations**

| Subgroup | Treatment | | |
|---|---|---|---|
| | A | B | C |
| 1 | 0 | 0 | 0 |
| 2 | 1 | 0 | 0 |
| 3 | 0 | 1 | 0 |
| 4 | 0 | 0 | 1 |
| 5 | 1 | 1 | 0 |
| 6 | 1 | 0 | 1 |
| 7 | 0 | 1 | 1 |
| 8 | 1 | 1 | 1 |

other than that/those involved in the particular hypothesis under test.

(a) When considering questions (i) and (ii) analyzing the full dataset we fit the model:

$$Y_i = \beta_0 + \beta_A A_i + \beta_B B_i + \beta_C C_i + \varepsilon_i$$

For question (i) we will test the significance of $\beta_A$, $\beta_B$ and $\beta_C$; for question (ii) we examine the contrasts $\beta_A$-$\beta_B$, $\beta_A$-$\beta_C$ and $\beta_B$-$\beta_C$.
When employing the full data set to consider the first order interactions between two treatments – question (iii) –we would fit the model including all these interactions:

$$Y_i = \beta_0 + \beta_A A_i + \beta_B B_i + \beta_C C_i + \beta_{AB} A_i B_i + \beta_{AC} A_i C + \beta_{BC} B_i C_i + \varepsilon_i.$$

We would then test the significance of $\beta_{AB}$, $\beta_{AC}$ and $\beta_{BC}$. For completeness we would also fit the second order interaction between A, B & C using the model below, although the study is not powered for that.

$$Y_i = \beta_0 + \beta_A A_i + \beta_B B_i + \beta_C C_i + \beta_{AB} A_i B_i + \beta_{AC} A_i C + \beta_{BC} B_i C_i + \beta_{ABC} A_i B_i C_i + \varepsilon_i.$$

(b) The subset of data used to fit the main effect of treatment A (say) would include only subgroups 1 and 2 and fit the model:

$$Y_i = \beta_0 + \beta_A A_i + \varepsilon_i.$$

To compare treatment A with B excluding interference from C we would use subgroups 1–3 and 5 and fit the model:

$$Y_i = \beta_0 + \beta_A A_i + \beta_B B_i + \varepsilon_i$$

Finally, to examine the interaction between treatment A and B excluding interference from C we would employ the same subgroups (1–3 & 5) and fit the model:

$$Y_i = \beta_0 + \beta_A A_i + \beta_B B_i + \beta_{AB} A_i B_i + \varepsilon_i.$$

The primary outcome measurement (thymic index, TI) will be made on infants of different sizes and ages. We will therefore carry out the analysis on the logarithm of the TI measurements since the (a) percentage changes are more appropriate, (b) the standard deviation is then essentially independent of the mean and (c) the residual distribution is more symmetrical. Otherwise the analysis will be based on least-squares regression controlling for infant size and season of observation and other covariates that have a noticeable effect on the variance.

Since three treatments or three pairs of treatments are under consideration for each of the above questions we have applied a Bonferroni correction for the best of three tests.

Table 6 gives the required numbers to achieve a power of 80% with significance level of 5%. The different columns correspond to the different questions listed above. The effect sizes considered are similar to those previously observed between seasons [8]. We also assume that between individuals the sd[log(TI)] = 0.21, which is the standard deviation of the residuals after controlling for infant size and season of observation derived from Collinson et al's data [8].

## Discussion

The ENID Trial has been designed to investigate the impact of combined pre- and post-natal nutritional supplementation on immune development in Gambian infants. The trial design will specifically allow us to establish which combination of supplements is most effective and also the most critical periods of intervention in pregnancy and

**Table 6 Sample size required for a range of effect sizes and different hypotheses to be tested**

| | (i) Main effects of treatments versus placebo | | (ii) Comparison of pairs of main effects versus one another | | (iii) Interactions between pairs of treatments | |
|---|---|---|---|---|---|---|
| Effect size (% difference in TI) | Full dataset | Reduced dataset* | Full dataset | Reduced dataset* | Full dataset | Reduced dataset* |
| 5% | 847 | 3388 | 1694 | 3388 | 3388 | 6776 |
| 7.5% | 385 | 1542 | 771 | 1542 | 1542 | 3084 |
| 10% | 222 | 888 | 444 | 888 | 888 | 1776 |

* Avoiding interference from irrelevant treatment.

infancy, and whether any observed effects are additive. To date, there are no previous registered or published trials on this topic. Previous trials of MMN [23] and PE [24] interventions in pregnancy have concentrated almost exclusively on birth weight and perinatal survival as outcomes. A single trial (in HIV-infected mothers) has investigated a limited range of T-cell subsets in pregnancy, but not in the offspring, and found that limited MMN supplementation (vitamins B, C and E), but not vitamin A alone, increased CD4+ and CD8+ counts [25]. The ENID Trial will also provide valuable data on other important developmental markers including growth.

Where there is evidence of benefit from a specific intervention/combination of interventions, future research should focus on refining the supplements to achieve the optimal, most cost-effective balance of interventions for improved health outcomes. Findings may also precipitate a separately funded larger or multi-centre trial with morbidity and/or mortality as the primary outcome. Where evidence indicates a defect in a specific component/components of immune function, more detailed interrogation within a subset of subjects from follow-up trials, likely to involve more intense sample collection schedules, will be justified, and will help to further our understanding of specific interactions. In the event that none of the supplementation combinations under trial have a marked impact on immune development across the whole cohort, data obtained may help pinpoint specific vulnerable groups (such as women with low pre-pregnancy BMI's for whom intervention may be most efficacious), for further targeted research.

#### Competing interests
The authors declare that they have no competing interests.

#### Authors' contributions
SEM, AJCF, MKD, LMJ, MLJ, & AMP all contributed to the development of the trial protocol. SEM drafted this manuscript and all authors reviewed critically for content and approved the final manuscript.

#### Acknowledgements
This trial is supported through by the Medical Research Council (UK), through core funding to the MRC International Nutrition Group (MC-A760-5QX00).

Received: 15 February 2012 Accepted: 31 August 2012
Published: 11 October 2012

#### References
1. Barker DJP: *Mothers, Babies and Diseases in Later Life*. London: BMJ Publishing Group; 1994.
2. Moore SE, Collinson AC, Tamba N'gom P, Aspinall R, Prentice AM: **Early immunological development and mortality from infectious disease in later life.** *Proc Nutr Soc* 2006, **65**(3):311–318.
3. Prentice AM, Whitehead RG, Roberts SB, Paul AA: **Long-term energy balance in childbearing Gambian women.** *Am J Clin Nutr* 1981, **34**:2790–2799.
4. Rayco-Solon P, Fulford AJ, Prentice AM: **Maternal preconceptional weight and gestational length.** *Am J Obstet Gynecol* 2005, **192**(4):1133–1136.
5. Rayco-Solon P, Fulford AJ, Prentice AM: **Differential effects of seasonality on preterm birth and intrauterine growth restriction in rural Africans.** *Am J Clin Nutr* 2005, **81**(1):134–139.
6. Moore SE, Cole TJ, Collinson AC, Poskitt EME, McGregor IA, Prentice AM: **Prenatal or early postnatal events predict infectious deaths in young adulthood in rural Africa.** *Int J Epidemiol* 1999, **28**:1088–1095.
7. Moore SE, Cole TJ, Poskitt EME, Sonko BJ, Whitehead RG, McGregor IA, *et al*: **Season of birth predicts mortality in rural Gambia.** *Nature* 1997, **388**:434.
8. Collinson AC, Moore SE, Cole TJ, Prentice AM: **Birth season and environmental influences on patterns of thymic growth in rural Gambian infants.** *Acta Paediatr* 2003, **92**(9):1014–1020.
9. Moore SE, Prentice AM, Wagatsuma Y, Fulford AJ, Collinson AC, Raqib R, *et al*: **Early-life nutritional and environmental determinants of thymic size in infants born in rural Bangladesh.** *Acta Paediatr* 2009, **98**(7):1168–1175.
10. Collinson AC, Ngom PT, Moore SE, Morgan G, Prentice AM: **Birth season and environmental influences on blood leucocyte and lymphocyte subpopulations in rural Gambian infants.** *BMC Immunol* 2008, **9**:18.
11. N'Gom PT, Collinson AC, Pido-Lopez J, Henson SM, Prentice AM, Aspinall R: **Improved thymic function in exclusively breastfed infants is associated with higher interleukin 7 concentrations in their mothers' breast milk.** *Am J Clin Nutr* 2004, **80**:722–728.
12. McDade TW, Beck MA, Kuzawa C, Adair LS: **Prenatal undernutrition, postnatal environments, and antibody response to vaccination in adolescence.** *Am J Clin Nutr* 2001, **74**(4):543–548.
13. Moore SE, Jalil F, Ashraf R, Szu SC, Prentice AM, Hanson LA: **Birth weight predicts response to vaccination in adults born in an urban slum in Lahore, Pakistan.** *Am J Clin Nutr* 2004, **80**:453–459.
14. Moore SE, Jalil F, Szu SC, Hahn-Zoric M, Prentice AM, Hanson LA: **Revaccination does not improve an observed deficit in antibody responses in Pakistani adults born of a lower birth weight.** *Vaccine* 2008, **26**(2):158–165.
15. Adu-Afarwuah S, Lartey A, Brown KH, Zlotkin S, Briend A, Dewey KG: **Randomized comparison of 3 types of micronutrient supplements for home fortification of complementary foods in Ghana: effects on growth and motor development.** *Am J Clin Nutr* 2007, **86**(2):412–420.
16. Briend A, Solomons NW: **The evolving applications of spreads as a FOODlet for improving the diets of infants and young children.** *Food Nutr Bull* 2003, **24**(3 Suppl):S34–S38.
17. Kaestel P, Michaelsen KF, Aaby P, Friis H: **Effects of prenatal multimicronutrient supplements on birth weight and perinatal mortality: a randomised, controlled trial in Guinea-Bissau.** *Eur J Clin Nutr* 2005, **59**(9):1081–1089.
18. Dubowitz LM, Dubowitz V, Palmer P, Verghote M: **A new approach to the neurological assessment of the preterm and full-term newborn infant.** *Brain Dev* 1980, **2**(1):3–14.
19. Albernaz E, Victora CG, Haisma H, Wright A, Coward WA: **Lactation counseling increases breast-feeding duration but not breast milk intake as measured by isotopic methods.** *J Nutr* 2003, **133**(1):205–210.
20. Haider R, Ashworth A, Kabir I, Huttly SR: **Effect of community-based peer counsellors on exclusive breastfeeding practices in Dhaka, Bangladesh: a randomised controlled trial [see commments].** *Lancet* 2000, **356**(9242):1643–1647.
21. WHO Motor Development Milestones. [cited 2012 14th February]; Available from: http://www.who.int/childgrowth/standards/motor_milestones/en/index.html.
22. Hasselbalch H, Nielsen MB, Jeppsen D, Pedersen JF, Karkov J: **Sonographic measurement of the thymus in infants.** *Eur Radiol* 1996, **6**:700–703.
23. Haider BA, Bhutta ZA: **Multiple-micronutrient supplementation for women during pregnancy.** *Cochrane Database Syst Rev* 2006, **4**:CD004905.
24. Kramer MS, Kakuma R: **Energy and protein intake in pregnancy.** *Cochrane Database Syst Rev* 2003, **4**:CD000032.
25. Fawzi WW, Msamanga GI, Spiegelman D, Wei R, Kapiga S, Villamor E, *et al*: **A randomized trial of multivitamin supplements and HIV disease progression and mortality.** *N Engl J Med* 2004, **351**(1):23–32.

doi:10.1186/1471-2393-12-107
**Cite this article as:** Moore *et al.*: A randomized trial to investigate the effects of pre-natal and infant nutritional supplementation on infant immune development in rural Gambia: the ENID trial: Early Nutrition and Immune Development. *BMC Pregnancy and Childbirth* 2012 **12**:107.
