# Supplementary material for: Impact of nutritional supplementation during pregnancy on antibody responses to diphtheria-tetanus-pertussis vaccination in infants: A randomised trial in The Gambia
Source: PLoS Med. 2019 Aug 6;16(8):e1002854. doi: 10.1371/journal.pmed.1002854 (PMC6684039; doi:10.1371/journal.pmed.1002854)
Supplement: S3 Appendix — (DOC) [file pmed.1002854.s003.doc]

# Medical Research Council Laboratories, Fajara

**Application to undertake a research project**

**__________________________________________________________________________**

#### A Summary Information

**A1 Title of project**

Please choose a short clear title for ease of reference and identification in future.

A randomized trial to investigate the effects of pre-natal and infancy nutritional supplementation on infant immune development

**A2 SCC Number** 1126

# Is this a resubmission? Yes

For ALL submissions and resubmissions a version number and version date needs to be entered below. Resubmissions may not be considered without this information

**VERSION NUMBER** 02

**Date of this version** July 21, 2008

**A3 Investigators (Principal Investigator first)**

Please list all investigators and collaborators and attach CV if the principal investigator is unknown to the Committee

| **Name** | **Institution** | **Position** |
| --- | --- | --- |
|  |  |  |
| Dr Sophie Moore | MRC Keneba | Head of Station |
| Dr Momodou Darboe | MRC Keneba | Higher Scientific Officer |
| Mrs Fatou Sosseh | MRC Keneba | Head Midwife |
| Dr Patrick Nshe | MRC Keneba | Medical Officer |
| Dr Pa Tamba N’Gom | MRC Fajara | Higher Scientific Officer |
| Dr Tony Fulford | LSHTM/MRC Keneba | Statistician |
| Dr Stefan Unger | MRC Keneba | Paediatric Registrar |
| Mr Amat Bah | National Nutrition Agency | Nutritionist |
| Prof Yukiko Wagatsuma | University of Tsukuba, Japan | Professor of Epidemiology |
| Dr Katie Flanagan | MRC Fajara | Infant Immunologist |
| Prof Andrew Prentice | LSHTM/MRC Keneba | Head, Nutrition Programme |
|  |  |  |

**Who will introduce the proposal at SCC?**

The PI, if present in The Gambia will normally be invited to present the proposal to the meeting

Dr Sophie Moore

**A4 Location(s) of research**

Please list all the places where the research will take place including field sites or health facilities

MRC Keneba and all villages covered by the West Kiang Demographic Surveillance System

**A5 Proposed start date and duration in months**

Start date: October 2008 for 48 months (see Appendix I for details).

**A6 Summary of project, long term objectives and specific aims (not more than 200 words)**

This section is very helpful to the Committees in determining quickly the main features of the study, and should be as clear and concise as possible. It should cover the key objectives and endpoints and, if the project is hypothesis driven, then the hypothesis should be stated here.

Infectious diseases continue to be the main cause of morbidity and mortality among infants and young children in LDCs. Both acute and chronic deficiencies of macro- and micro-nutrients result in an impairment to a number of components of the immune system with the assumption that this inter-relationship between nutritional deficiency and immunity may underlie disease susceptibility. Recent research from our group has specifically focused on the hypothesis that nutritional status during fetal life and early infancy may be critical for immune development, and have long-lasting effects on functional immunity. Data from The Gambia, Pakistan and Bangladesh provides support for this hypothesis, with associations observed between indicators of early nutritional exposures and later immune function. Whilst these data highlight the potential importance of both pre- and post-natal nutritional status for the optimal development of the human immune system, they are all observational in nature. Little is known about the impact and mechanisms of action of nutritional supplementation on infant immune development and for targeted therapeutic nutrition to be of greatest benefit, further research in this area is required.

We are proposing a randomised control trial of combined pre- and post-natal nutritional supplementation among pregnant women and their infants in the West Kiang region of The Gambia. The aim of this study is to determine the impact and potential mechanisms of different supplementation regimens on infant immune development. The primary outcome of this study will be thymic development during infancy, with antibody response to vaccination and lymphocyte function assessed as secondary functional outcomes.

**A7 Confidentiality**

SCC applications will normally be available on the MRC Gambia intranet with access for all senior staff. If for reasons of commercial, ethical or scientific sensitivity you wish to restrict access/circulation to SCC Committee members only please indicate here.

**Restrict access to SCC members?** No

**A8 Checklist/Signatures**

**Please complete the following checklist and comment as appropriate.** This section is designed to ensure that all the planning steps have been taken that are needed for a successful project and that the resource requirements are appropriately laid out in Section D. For projects at the MRC Laboratories, Programme Heads will help visiting workers, and others preparing proposals at a distance, to ensure liaison with key individuals who need to be consulted locally.

1. **Has the project been discussed and cleared with the institutions in which research will be carried out including health services to which the study will need access?**

Yes, the proposed project has been discussed with all key members of the MRC’s Nutrition Programme, and with the administration at the Keneba field site. The Regional Health Team at Mansakonko have been consulted, and their approval for the study given.

1. **Have all investigators and collaborators given their agreement to take part in the study as described?**

Yes, all investigators and collaborators have approved this final version of the study application and given their agreement to take part.

1. **Have ethical issues been addressed?** Give details in section C**.**

Yes, see section C.

1. **Does the project require laboratory work, new laboratory procedures, or the riding of motorcycles? Have the safety issues been addressed?** (The Health and Safety Manager will advise on risk assessment)

The project will require laboratory work, although no new laboratory procedures will be introduced; existing laboratory safety procedures will be adhered to.

The project will be set up according to GCP requirements, with the trial registered and a trial monitor and data safety monitor appointed.

The project will require substantial use of motorcycles. All safety issues will be addressed, including the provision of helmets, boots and safety clothing to staff riding motorcycles.

1. **Will the project require data and/or materials to be taken out of The Gambia? If so please give details and sign the following statements:**

Aliquots of plasma and breast milk will be sent to MRC Human Nutrition Research, Cambridge for the assessment of micronutrient levels.

A complete copy of the final, cleaned data set will remain with the data centre at MRC Keneba. A copy of the data may later be transferred to LSHTM for statistical analysis by the project statistician, Dr Tony Fulford.

The principal investigator undertakes to leave with the Computer Centre data archivist in The Gambia, a complete copy of the data set at the following two time points:

1. After data entry and verification (“raw data sets”)
2. At the point of submission for publication of final report (“analysis data sets”)

**Signed:
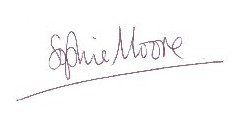
**

The principal investigator undertakes to leave with MRC Laboratories The Gambia appropriate aliquots of the biological material being taken out of the country

**Signed:
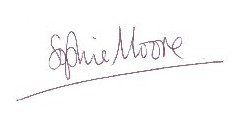
**

1. **For projects to be carried out at MRC Laboratories:**

**Has the project been discussed with the following support staff as appropriate and resource requests agreed? (Give details where relevant in section D) :**

|  | DATE DISCUSSED | Comment |
| --- | --- | --- |
| Health and Safety Manager | June 2008 | Discussed with Dr Landing Jarjou. |
| Head of IT/Senior Data Manager | June 2008 | Discussed with Dr Tony Fulford & Mr Jahid Hasan. |
| Director of Clinical Services | June 2008 | Discussed locally in Keneba & with Lower River Region Regional Health Team. |
| Scientific Administrator | June 2008 | Approved by Prof Andrew Prentice. |
|  |  |  |
| Transport Manager | June 2008 | Transport requirements discussed locally. |
| Finance Manager | June 2008 | Approved by Prof Andrew Prentice. |
| Personnel Manager | June 2008 | Staff requirements discussed locally. |
| Director of Operations | June 2008 | Approved by Prof Andrew Prentice. |
| Other services – specify  Laboratory Manager  Trial Monitoring/GCP | June 2008  June 2008 | Discussed with Mr Mamodou Bah.  Discussed with Dr Jenny Mueller. |

**Signature of principal investigator:
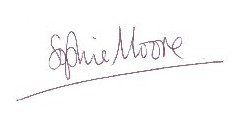
Date:** July 21, 2008

**________________________________________________________________________**

**B Description of Project**

not more than 5 pages covering the following:

**B1 Background**

The background should show the relationship between the proposed study and the present state of knowledge and should reference previous work by the investigators and others. The results of any pilot experiments should be stated.

The inter-relationship between nutritional status and immune function continues to be the focus of much research and debate [1, 2]. It is well documented that acute and chronic deficiency of both macro- and micro-nutrients results in an impairment to a number of components of the immune system [3] and supplementation with individual micronutrients has proven efficacious as therapy for certain infectious morbidities, including vitamin A and measles infection [4], and zinc and diarrhoeal disease [5]. Conversely however, more recent research also suggests that supplementation with specific micronutrients may have non-specific deleterious effects on immune function, with iron [6] and vitamin A [7] implicated. Further work to understand the mechanism of these effects is required.

In addition to the effects of contemporaneous nutritional status on human immune function, recent evidence from our group and others suggests that nutritional status during fetal life and early infancy may be critical for immune development, with long lasting consequences. A prospective birth-cohort study of neonatal immune function and development in West Kiang demonstrated seasonal effects on thymic size, with smallest thymuses found in infants both born and when measured in the hungry/wet season, irrespective of infant weight [8]. In the same cohort, lymphocyte subpopulation counts throughout the first year of life were significantly affected by season of birth [9]. Furthermore, seasonal variation was also observed in the frequency of signal joint T-cell receptor rearrangement excision circles (sjTRECs), which provide an indirect assay of thymus function [10]. Breast milk levels of IL-7, a cytokine critical for thymic and T-cell development, were significantly lower in samples collected in the hungry/wet season, suggesting a possible post-natal influence on thymic development [10]. Our observation of an effect of early-life exposures on thymic development in The Gambia has been replicated in a large study of infants from rural Bangladesh: birth weight was positively correlated with thymic size up to 8 wk of age and a strong effect of month of measurement was observed at all time points (Moore *et al*, submitted). These infants are currently being followed up to investigate the long-term sequelae of early life effects on thymic development.

Existing data to support longer-term functional consequences to immunity includes studies from the Philippines and Pakistan that demonstrate a positive association between birth weight and antibody response to vaccination in adolescence [11] and adulthood [12, 13]. In a cohort of adults born in an urban slum in Lahore, Pakistan, we observed a positive association between birthweight and antibody response to a Vi polysaccharide vaccine for *Salmonella typhi* [12]. To investigate this further, we revaccinated these subjects with a second dose of the same Vi vaccine and also administered a polysaccharide-protein conjugate vaccine (conjugated *Haemophilus influenzae* type b (Hib) vaccine). Anti-Vi IgG levels remained positively correlated with birthweight but no associations were observed between anti-Hib IgG levels and size at birth [13].

Together, these data highlight the importance of both pre- and post-natal nutritional status for the optimal development of the human immune system and support the need for randomized intervention trials assessing the impact of nutritional supplementation on infant immune development.

**Supplementation in pregnancy**

In developing countries, nutritional deficiencies are common and are associated with poor pregnancy outcomes, which may in turn have longer-term effects on human health [14]. Many components of the immune system develop early in fetal life with, for example, functional lymphocytes appearing in the fetal circulation as early as 10-12 wk gestation [15]. As such, deficiencies of critical nutrients essential for development may have long lasting effects. A recently published series in The Lancet included two reviews specifically focusing on the impact of maternal and child undernutrition on survival [16] and long-term health [17]. Whilst a number of interventions were estimated to reduce infant mortality between birth and 36 months by about 25% [16], it was concluded that there is insufficient evidence currently available about the effects of early nutrition on long-term changes in immune function [17].

Current policy in The Gambia recommends a daily supplement of 60 mg iron and 400 μg folate (FeFol) to all pregnant women, with the primary aim of reducing iron deficiency anaemia. However, it is known that many women face additional macro- and micronutrient deficiencies, potentially compromising their health, pregnancy outcomes, and infant survival. An earlier study of nutritional supplementation during pregnancy in The Gambia randomised women to a daily supplement made from groundnuts, rice flour, sugar and groundnut oil. Two daily biscuits provided a maximum daily intake of 4250kJ energy, 22g protein, 56g fat, 47mg calcium and 1.8mg iron [18]. Mean birth weight in the supplementation group was increased by 136g (95% CI: 79, 193) overall, with a difference of 201g (95% CI: 132, 270) during the nutritionally poor ‘hungry’ season. Peri-natal mortality was also affected by supplementation status; the odds ratio of stillbirth in the intervention group was 0.47 (95% CI: 0.23, 0.99) and for deaths in the first week of life it was 0.54 (95% CI: 0.35, 0.85) [18]. Whilst no data are available on cause of death from this study, it is likely that the majority of early deaths in this age group were infection-related. Together, these data highlight the potential benefits of nutritional intervention during pregnancy and the current proposal will therefore incorporate a pre-natal supplementation arm, specifically designed to assess the added benefit of supplementary nutrition beyond the usual standard of care provided in The Gambia on infant immune development.

**Supplementation in infancy**

Recent attention has focused on the association between an infant’s rate of growth during early-infancy and later disease risk with, for example, faster rates of post-natal ‘catch-up’ growth implicated as a possible causative factor for certain chronic disease outcomes [19]. In countries such as The Gambia, poor growth in infancy results from a combination of the early introduction of nutritionally-poor complementary foods and frequent infectious disease episodes, but the long-term effects of this pattern are not fully understood. Growth faltering is most evident during the first phase of complementary feeding (Figure 1) when foods of low nutrient density begin to replace breast milk and rates of diarrhoeal illness caused by food contamination are at their highest.

Figure 1: Weight, length, head circumference and BMI Z-scores from birth to 52 weeks in West Kiang infants.

Data from Collinson et al [20]. Data from boys and girls combined. Error bars = SEM.

In addition to general nutritional deficiencies, micronutrient deficiencies are highly prevalent among infants because of their high nutrient needs relative to energy intake and the effects of frequent infection on appetite, nutrient absorption and nutrient losses [21]. Fortification (either processed complementary foods or home fortification) is the most feasible option in most circumstances, given the cost of most micronutrient rich foods. Fortification can help ensure micronutrient intakes when nutrient-rich local foods are costly or unavailable. A recent systematic review looked at the efficacy and effectiveness of complementary feeding interventions in developing country settings [21]. From a total of 10 intervention studies that included data on morbidity outcomes, beneficial effects were observed in diarrhoea and both upper and lower respiratory infections, although a number of smaller studies showed no impact. No studies have looked directly at the effects of infant immune development.

The WHO recommends exclusive breastfeeding to six months of age, a recommendation endorsed in The Gambia by the National Nutrition Agency (NaNA) and Department of State for Health and Social Welfare (DoSHSW). A community-based trial of nutritional supplementation before 6 months of age would not therefore be appropriate in this setting. It is well recognised that the period of complementary feeding, from 6 to 24 months of age, is one of the most critical times for preventing malnutrition. Nutritional supplementation during this period of development may therefore be of added benefit to infants and young children in this population setting.

**Summary**

We are proposing a randomized controlled trial of combined pre- and post-natal nutritional supplementation on infant immune development in the West Kiang region of The Gambia. During pregnancy, we will assess the additive value of supplemental nutrients given to women from 12 wk of gestation against the usual standard of care (FeFol only). From 6 mo of age, we will further randomize their infants to receive a fortified weaning food supplement or placebo. This study will test the primary hypothesis that combined pre-and post-natal supplementation will impact positively on infant immune development.

**B2 References**

1. G Morgan: What, if any, is the effect of malnutrition on immunological competence? *Lancet* 1997, 349:1693-1695.

2. UE Schaible, SH Kaufmann: Malnutrition and infection: complex mechanisms and global impacts. *PLoS Med* 2007, 4:e115.

3. NS Scrimshaw, JP SanGiovanni: Synergism of nutrition, infection, and immunity: an overview. *Am J Clin Nutr* 1997, 66:464S-477S.

4. GD Hussey, M Klein: Routine high-dose vitamin A therapy for children hospitalized with measles. *J Trop Pediatr* 1993, 39:342-5.

5. AH Baqui, RE Black, S El Arifeen, M Yunus, J Chakraborty, S Ahmed, JP Vaughan: Effect of zinc supplementation started during diarrhoea on morbidity and mortality in Bangladeshi children: community randomised trial. *BMJ* 2002, 325:1059.

6. S Sazawal, RE Black, M Ramsan, HM Chwaya, RJ Stoltzfus, A Dutta, U Dhingra, I Kabole, S Deb, MK Othman, et al: Effects of routine prophylactic supplementation with iron and folic acid on admission to hospital and mortality in preschool children in a high malaria transmission setting: community-based, randomised, placebo-controlled trial. *Lancet* 2006, 367:133-43.

7. CS Benn, AB Fisker, MJ Jorgensen, P Aaby: Why worry: vitamin A with DTP vaccine? *Vaccine* 2007, 25:777-9.

8. AC Collinson, SE Moore, TJ Cole, AM Prentice: Birth season and environmental influences on patterns of thymic growth in rural Gambian infants. *Acta Paediatr* 2003, 92:1014-20.

9. AC Collinson, PT Ngom, SE Moore, G Morgan, AM Prentice: Birth season and environmental influences on blood leucocyte and lymphocyte subpopulations in rural Gambian infants. *BMC Immunol* 2008, 9:18.

10. PT N'Gom, AC Collinson, J Pido-Lopez, SM Henson, AM Prentice, R Aspinall: Improved thymic function in exclusively breastfed infants is associated with higher interleukin 7 concentrations in their mothers' breast milk. *Am J Clin Nutr* 2004, 80:722-8.

11. TW McDade, MA Beck, C Kuzawa, LS Adair: Prenatal undernutrition, postnatal environments, and antibody response to vaccination in adolescence. *Am J Clin Nutr* 2001, 74:543-8.

12. SE Moore, F Jalil, R Ashraf, SC Szu, AM Prentice, LA Hanson: Birth weight predicts response to vaccination in adults born in an urban slum in Lahore, Pakistan. *Am J Clin Nutr* 2004, 80:453-459.

13. SE Moore, F Jalil, SC Szu, M Hahn-Zoric, AM Prentice, LA Hanson: Revaccination does not improve an observed deficit in antibody responses in Pakistani adults born of a lower birth weight. *Vaccine* 2008, 26:158-65.

14. S Owens, CH Fall: Consequences of poor maternal micronutrition before and during early pregnancy. *Trans R Soc Trop Med Hyg* 2008, 102:103-4.

15. A Whitelaw, J Parkin: Development of immunity. *British Medical Bulletin* 1988, 44:1037-1051.

16. ZA Bhutta, T Ahmed, RE Black, S Cousens, K Dewey, E Giugliani, BA Haider, B Kirkwood, SS Morris, HP Sachdev, et al: What works? Interventions for maternal and child undernutrition and survival. *Lancet* 2008, 371:417-40.

17. CG Victora, L Adair, C Fall, PC Hallal, R Martorell, L Richter, HS Sachdev: Maternal and child undernutrition: consequences for adult health and human capital. *Lancet* 2008, 371:340-57.

18. SM Ceesay, AM Prentice, TJ Cole, F Foord, LT Weaver, EME Poskitt, RG Whitehead: Effects on birthweight and perinatal mortality of maternal dietary supplementation in a primary health care setting in rural Gambia. *British Medical Journal* 1997, 315:786-790.

19. A Singhal, A Lucas: Early origins of cardiovascular disease: is there a unifying hypothesis? *Lancet* 2004, 363:1642-5.

20. A Collinson, S Moore, M O'Connell, C Charalambos, A Prentice: Developmental changes in leptin as a measure of energy status in human infants in a natural ecologic setting. *Am J Clin Nutr* 2005, 81:488-94.

21. KG Dewey, S Adu-Afarwuah: Systematic review of the efficacy and effectiveness of complementary feeding interventions in developing countries. *Matern Child Nutr* 2008, 4 Suppl 1:24-85.

22. P Kaestel, KF Michaelsen, P Aaby, H Friis: Effects of prenatal multimicronutrient supplements on birth weight and perinatal mortality: a randomised, controlled trial in Guinea-Bissau. *Eur J Clin Nutr* 2005, 59:1081-9.

23. E Albernaz, CG Victora, H Haisma, A Wright, WA Coward: Lactation counseling increases breast-feeding duration but not breast milk intake as measured by isotopic methods. *J Nutr* 2003, 133:205-10.

24. R Haider, A Ashworth, I Kabir, SR Huttly: Effect of community-based peer counsellors on exclusive breastfeeding practices in Dhaka, Bangladesh: a randomised controlled trial. *Lancet* 2000, 356:1643-7.

25. S Adu-Afarwuah, A Lartey, KH Brown, S Zlotkin, A Briend, KG Dewey: Randomized comparison of 3 types of micronutrient supplements for home fortification of complementary foods in Ghana: effects on growth and motor development. *Am J Clin Nutr* 2007, 86:412-20.

26. A Briend, NW Solomons: The evolving applications of spreads as a FOODlet for improving the diets of infants and young children. *Food Nutr Bull* 2003, 24:S34-8.

27. H Hasselbalch, MB Nielsen, D Jeppsen, JF Pedersen, J Karkov: Sonographic measurement of the thymus in infants. *European Radiology* 1996, 6:700-703.

28. P Aaby, C Marx, S Trautner, D Rudaa, H Hasselbalch, H Jensen, I Lisse: Thymus size at birth is associated with infant mortality: a community study from Guinea-Bissau. *Acta Paediatr* 2002, 91:698-703.

29. G Walraven, M Telfer, J Rowley, C Ronsmans: Maternal mortality in rural Gambia: levels, causes and contributing factors. *Bull World Health Organ* 2000, 78:603-13.

30. BA Haider, ZA Bhutta: Multiple-micronutrient supplementation for women during pregnancy. *Cochrane Database Syst Rev* 2006:CD004905.

### B3 Project description

This should cover project plan, time-scales, descriptions of methods, justification, analyses to be carried out, expected outcomes (see also B4 and B5 where particular details need to be set out and cross refer as necessary).

The proposed project is a factorial randomised controlled trial of nutritional supplementation to pregnant women and their infants in the West Kiang region of The Gambia. The primary outcome is infant thymic development, with antibody response to vaccination and lymphocyte function as secondary functional outcomes.

*Timescales:*

Preparation for the study is ongoing, with the active liaison with collaborators and key partners including the National Nutrition Agency (NaNA) and Regional Health Team (RHT), Mansakonko, Lower River Region. Following full SCC and EC approval, community sensitisation will begin with village meetings across West Kiang. Once local approval is granted, all eligible women of reproductive age will be contacted and invited to participate in the study. It is anticipated that recruitment of pregnant women (12 wk gestation) will commence in October 2008, and fieldwork for the full study will run until April 2012 (when all infants reach 12 months of age). A full study timeline is detailed in Appendix I.

*Project plan:*

**Pre-pregnancy and pregnancy protocol**

The study will be based in all 36 villages currently registered within the West Kiang Demographic Surveillance System (DSS); see Appendix II for village listing and DSS reported births for 2007. All women of reproductive age (18 to 45 years) will be invited to participate; present estimates from the DSS indicate the current population to include 2,440 women in this age group. Following approval from the local community, each woman identified will be visited by a member of the study team and a detailed explanation of the study will be given and informed consent obtained from those women expressing a willingness to participate. Each month from enrolment, participating women will be visited by a member of the study team with a short questionnaire on the date of their last menstrual period (LMP). Where two consecutive periods are missed, a urine sample will be collected for pregnancy testing. The study midwife will then inform women of the outcome of their pregnancy test, and women with a positive test will be invited to Keneba for an initial study visit (Visit 1). Based on the date of their LMP, this visit will be timed to coincide with approximately 12 wk of gestation. Details of planned measurements and samples to be collected at this visit and at each subsequent pre-natal visit are given in full later in this section.

Following this initial visit, all women with a confirmed pregnancy of 12 wk ±2 wk (as assessed by ultrasound) will be randomly assigned to one of four pre-natal supplements:

1. FeFol: Iron-folate, representing the usual standard of care during pregnancy, as per Gambian Government guidelines.
2. MMN: Multiple micronutrients. A combination of 15 micronutrients, specifically designed for use during pregnancy, and as formulated by UNICEF. A single tablet provides the Recommended Dietary Allowance (RDA) for each micronutrient. However, recent research from Guinea-Bissau suggests that a daily supplement of twice the RDA is more effective with regard to birth outcomes [22]. We will therefore supplement women in this arm of the trial with two daily MMN tablets, which will also ensure a comparable level of iron to the FeFol arm.
3. PE + FeFol: Protein-energy and iron-folate. A food-based supplement developed by Nutriset SA, Malaunay, France, providing a comparable level of iron and folate to the FeFol only arm, but with the addition of energy, protein and lipids.
4. PE + MMN: Protein-energy and multiple micronutrients. A micronutrient fortified food-based supplement also developed by Nutriset SA, and providing comparable levels of micronutrients to the MMN arm (including FeFol), in addition to the energy and protein and lipid content.

The composition of these supplements is as outlined in Table 1 below:

Table 1: Nutritional composition of pre-natal supplements

|  | **FeFol** | **MMN** | **PE+FeFol** | **PE+MMN** |
| --- | --- | --- | --- | --- |
| Iron (mg) | 60 | 60 | 60 | 60 |
| Folate (μg) | 400 | 400 | 400 | 400 |
| Vitamin A (μg) |  | 1600 |  | 1600 |
| Vitamin D (IU) |  | 400 |  | 400 |
| Vitamin E (mg) |  | 20 |  | 20 |
| Vitamin C (mg) |  | 140 |  | 140 |
| Vitamin B1 (mg) |  | 2.8 |  | 2.8 |
| Vitamin B2 (mg |  | 2.8 |  | 2.8 |
| Niacin (mg) |  | 36 |  | 36 |
| Vitamin B6 (mg) |  | 2.8 |  | 2.8 |
| Vitamin B12 (μg) |  | 5.2 |  | 5.2 |
| Zinc (mg) |  | 30 |  | 30 |
| Copper (mg) |  | 4 |  | 4 |
| Selenium (μg) |  | 130 |  | 130 |
| Iodine (μg) |  | 300 |  | 300 |
| Energy (kcal) |  |  | 746 | 746 |
| Protein (g) |  |  | 20.8 | 20.8 |
| Lipids (g) |  |  | 52.6 | 52.6 |
|  |  |  |  |  |

Each supplement will be administered to the women on a daily basis by field assistants posted to the community and assisted by village assistants. Subject compliance to the supplement will therefore be recorded on a daily basis. This arm of the trial will be open, since it will not be possible to blind the field assistants or the women to the supplement type; all other investigators however will not know to which group the women belong. Maternal morbidity will be assessed by questionnaire, administered by the field assistants on a weekly basis. Women requiring medical attention, will be visited by a trekking nurse, and referred to the clinic at MRC Keneba if required. At 20 and 30 wk gestation, women will be invited to Keneba for two further antenatal assessments, as detailed below.

*Scheduled pre-natal measurements and sample collection details (12, 20 & 30 wk gestation):*

Standard antenatal check: including blood pressure; haemoglobin; urine sample. Women will also be offered HIV testing, as is routine practice in West Kiang.

Maternal anthropometry: weight; standing height; sitting height; waist & hip circumference; mid-upper-arm circumference; and body composition. All using standard, regularly validated equipment and following standard operating procedures (SOPs).

Venous blood sample: haematology and plasma micronutrient status.

Fetal ultrasound: Fetal growth parameters by ultrasound, including bi-parietal diameter, abdominal circumference and femur length.

**Delivery protocol**

It is our intention that all deliveries occurring in West Kiang will be attended by a trained field assistant. It will not be possible for us to attend women who deliver outside West Kiang, although they will be followed up and, where possible, included in the remainder of the study. We will work closely with the West Kiang Traditional Birth Attendants (TBAs) who will notify village assistants when a woman goes into labour. The field assistant responsible for that village will be contacted and be available for sample collection following delivery. This system worked extremely well for our recently completed trial of peri-conceptual multiple micronutrients (SCC 1000; PI Dr Stephen Owens), with excellent cooperation from TBAs and the community.

Following delivery a sample of cord blood (10mL) will be collected and the placenta will be trimmed and weighed according to standard protocols; all samples will then be transported to the Keneba lab for processing. Cord blood plasma and serum will then be stored for assessment of micronutrient levels and a small full-thickness biopsy taken from the placenta, washed and re-suspended in formalin for later histological analysis.

Within 72 hours of delivery, all women and their newborn infants will be visited by the study midwife for a ‘baby check’. This is a routine practice for the three core study villages (Keneba, Kantong Kunda & Manduar), which includes infant anthropometry (weight, length, MUAC, head circumference), gestational age assessment and a general health check. Infants assessed as unwell will be referred to the MRC Keneba clinic for assessment by the study clinician/resident paediatrician.

**Infancy protocol**

In The Gambia, exclusive breastfeeding to six months of age is recommended and the National Nutrition Agency (NaNA) actively promotes this practice to the population. Community-based peer counselling in exclusive breastfeeding has proven efficacious in improving both the rate and duration of exclusive breastfeeding [23, 24]. In the proposed study, we will work with NaNA to provide exclusive breastfeeding counselling to all participating women. This will include one-to-one sessions with trained personnel both before and after delivery, where the importance of exclusive breastfeeding will be discussed along with a discussion of the benefits of the continuation of breast feeding beyond the period when complementary foods are introduced. The purpose of this element of the study is to ensure that all women are provided with the knowledge to provide optimal nutrition to their infants during the period from birth to 6 months of age.

From 6 months of age, infants will be randomised to receive a nutrient enriched weaning food fortificant or placebo. In a recent trial in Ghana, 3 types of micronutrient supplements for home fortification of complementary foods were compared [25]. The most effective supplement was found to be an energy-dense peanut-based fortified spread developed by Nutriset SA, Malaunay, France (‘Nutributter’, [26]). We will use a similar weaning food fortificant as used in the Ghana trial or an unfortified placebo; both products will be produced by Nutriset.

The nutritional composition of the fortificant is as detailed in Table 2:

Table 2: Nutritional composition of 20g of weaning food fortificant

| **Nutrient** | **Amount per 20g** | **Nutrient** | **Amount per 20g** |
| --- | --- | --- | --- |
| β-Carotene (μg RE) | 400 | Potassium (mg) | 152 |
| Vitamin C (mg) | 30 | Copper (mg) | 0.2 |
| Folic acid (μg) | 80 | Selenium (μg) | 10 |
| Thiamine (mg) | 0.3 | Iodine (μg) | 90 |
| Riboflavin (mg) | 0.4 | Phosphorus (mg) | 82 |
| Vitamin B3 (mg) | 4 | Magnesium (mg) | 16 |
| Pantothenic acid (mg) | 1.8 | Manganese (mg) | 0.08 |
| Vitamin B6 (mg) | 0.3 | Total energy (kcal) | 108 |
| Vitamin B12 (μg) | 0.5 | Linoleic acid (g) | 1.29 |
| Iron (mg) | 9 | Linolenic acid (g) | 0.29 |
| Zinc (mg) | 4 | Phytate (mg) | 82 |
| Calcium (mg) | 100 |  |  |
|  |  |  |  |

Infants in the placebo arm of the study will receive an identically packaged formulation, of minimal nutrient value. The supplement will be administered in foil wrapped packets and given to the mothers on a weekly basis. The mother will be asked to administer the supplement/placebo on a daily basis by mixing the full amount with 1-2 tablespoons of the infant’s normal weaning food. Mothers will be encouraged to give the study infant the full dose, and compliance will be assessed by both unannounced observations and by a questionnaire administered on a weekly basis. At these weekly visits, a morbidity questionnaire will also be completed. Monthly 24-h recalls will be performed to assess the infant’s dietary intake.

*Scheduled infancy measurements and sample collection details:*

Following delivery, infants will be seen at the Keneba field site at 1, 8, 24 & 52 wk of age for assessment of the primary outcome, thymus size. Thymus size will be assessed sonographically using a validated method in which the transverse diameter of the thymus and the saggital area of its largest lobe are multiplied to give a volume-related thymic index (TI) [27]. This index has been shown to correlate with thymus weight at autopsy and has been used previously by our group both in Keneba and in Bangladesh to show that the human thymus is sensitive to environmental influences during infancy [8]. Of note, a study from Guinea Bissau indicated that a small thymus at birth predicts an increase in risk of infection-related mortality in infancy [28]. Thymus size will be measured using a Siemens Acuson ultrasound unit together with a P10-4 Transducer (Siemens, UK).

Secondary outcomes in the study will include antibody response to vaccination, lymphocyte subsets and function, as follows.

Antibody response to vaccination (12, 24 & 52 wk of age):

Anti-diphtheria, tetanus toxoid and Hib antibody titres will be measured using standard methodology (100 μL serum/antibody).

Lymphocyte subset phenotype enumeration (up to 7 colours) (24 & 52 wk of age):

We will use two 7 colour panels to assess for naïve effector/central memory and activation/differentiation cells (100μL whole blood/panel), which will include the following markers:

- Panel 1: CD3, CD4, CD8, CD27, CD57, CD28, CD44, CD45RA/RO.
- Panel 2: CD3, CD4, CD8, CD62L, CD38, HLADR.

Lymphocyte function (24 & 52 wk of age):

Whole blood will be stimulated overnight with test antigens (100μL/antigen, to include a positive control antigen, e.g. anti-CD3/antiCD28 or PHA, vaccine antigens, e.g. PPD, HBV, TT and streptococcus) and cell proliferation and cytokine production (to include IL-6, TNF-α, IFN-γ, IL-2, IL-13, IL-10) assessed by thymidine incorporation and luminex cytokine assessement.

At these time points (1, 8, 12, 24 & 52 wk of age), we will also measure infant anthropometry and body composition and collect a small sample of breast milk (5mL from each breast) from each lactating woman, for the assessment of breast milk micronutrient levels. All infants will also be seen at the additional time points for administration of the EPI vaccines, as per Gambian government protocol (Table 3). For subjects outside of the core villages (Keneba, Kantong Kunda and Manduar), administration of EPI vaccines will be co-ordinated with the RHT trekking team.

Table 3: EPI vaccine schedule – The Gambia

| **Age (wk)** | **Vaccine** |
| --- | --- |
| 0 (within 72 hours of birth) | BCG, HBV, OPV |
| 4 | OPV |
| 8 | DTP, Hib, HBV, OPV |
| 12 | DTP, Hib, OPV |
| 16 | DTP, Hib, HBV, OPV |
| 40 | Measles, Yellow Fever, OPV |

If subjects consent, both maternal and infant residual blood cells will be stored and DNA extracted for later genetic analysis. Once extracted, DNA will be banked and used according to the Gambian initiative for DNA collections.

**B4 Details of study design and investigations**

This section is designed to give the Committees sufficient information to see clearly and quickly the scientific and ethical aspects of the study design. Some parts will not be relevant to all studies. For studies at MRC Laboratories, the please discuss data management arrangements with the Head of Computing. For clinical studies, please discuss the clinical service commitments with the Clinical Director. If you have questionnaires or consent forms prepared, please attach these to the application.

1. **What type of study design is proposed (eg case control, prospective cohort, randomised controlled trial, etc)**

Three-way randomised controlled trial.

1. **What is the proposed size of the study (this may relate to patients, cases, controls, survey subjects, laboratory samples etc, as appropriate).**

800 mother-infant pairs. We anticipate about 1000 births over a two year period and a 80% participation rate.

1. **Please describe the statistical considerations and sample size calculations involved in determining the size of the study.** (If you do not have access to statistical advice, please consult the MRC Laboratories Statistics Department.)

There are three types of questions that the proposed study will address:

(i) Main effects due to each supplement.

(ii) Comparison of main effects of different supplements.

(iii) Effect modification of one supplement by another.

In each case we consider analysis of both (a) the full data set and (b) also in the subset of data for which the control is given for the treatment not involved in the analysis (this is in order to remove the potential interpretational complications of interactions between treatments).

The primary outcome measurements (thymic index, TI) will be made on infants of different sizes and ages. We will therefore carry out the analysis on the logarithm of the TI measurements since the (a) percentage changes are more appropriate, (b) the standard deviation is then essentially independent of the mean and (c) the residual distribution is more symmetrical. Otherwise the analysis will be based on least-squares regression controlling for infant size and season of observation and other covariates that have a noticeable effect on the variance. A random effects model will be used to account for any potential clustering effects by village of residence.

Since three treatments or three pairs of treatments are under consideration for each of the above questions we have applied a Bonferroni correction for the best of three tests.

The following table gives the required numbers to achieve a power of 80% with significance level of 5%. The different columns correspond to the different questions listed above. The effect sizes considered are of the similar to those previously observed between seasons [8]. We also assume that between individuals the sd[log(TI)]=0.21, which is the standard deviation of the residuals after controlling for infant size and season of observation derived from Collinson et al’s data [8].

Table 4 Sample size required for a range of effect sizes and different hypotheses to be tested.

|  | Main effects | | Comparison of pairs of main effects | | Interactions between pairs of treatments | |
| --- | --- | --- | --- | --- | --- | --- |
| Effect size | (i)a | (i)b | (ii)a | (ii)b | (iii)a | (iii)b |
| 5% | 847 | 3388 | 1694 | 3388 | 3388 | 6776 |
| 7.5% | 385 | 1542 | 771 | 1542 | 1542 | 3084 |
| 10% | 222 | 888 | 444 | 888 | 888 | 1776 |

**For studies involving human subjects:**

1. **How and where will the study subjects (cases, controls, etc) be selected? Has it been confirmed that they are not already involved in other studies?**

Subjects will be recruited using the West Kiang Demographic Surveillance System; all women resident in West Kiang and aged between 18 and 45 y of age will be invited to participate. A small number of young women (aged 21 to 24 y) are participating in an ongoing longitudinal investigation of the effects of adolescence on skeletal response to pregnancy and lactation in the mother on bone development in the infant, SCC 998. These women will be excluded from the current protocol.

1. **What inclusion/exclusion criteria will be applied?**

Inclusion criteria:

Women: Resident in West Kiang and aged between 18 – 45 y at August 1^st^ 2008.

Planning to remain resident in West Kiang for the next 36 months.

Infants: All infants born to women enrolled into the pre-natal arm of the study

Exclusion criteria:

Women: Currently enrolled in another MRC study

Known to be asthmatic or allergic to peanuts

Current pregnancy (beyond 12 wk on ultrasound assessment)

Severe anaemia (haemoglobin < 7 g/dL)

Known sickle cell disease

Reported onset of menopause

Infants: Major congenital malformations

Known to be asthmatic or allergic to peanuts

Severe malnutrition (weight-for-height Z-score < -3)

1. **What samples, if any, will be taken and what investigations will be conducted?**

As in text above, but Appendix III also outlines the sample collection schedule and details in full.

1. **Will treatment be given?** Yes.

**If yes:**

**Nature of treatment(s)**

**For drugs: dosage and duration of treatment**

Pregnant women from 3 months gestation to delivery, nutritional supplementation as detailed in Table 1. All women will also receive two doses of intermittent presumptive treatment (IPT), for the prevention of malaria, as recommended by the Gambian Government.

Infants from 6 to 12 months of age, weaning food fortificant, or placebo, as detailed in Table 2.

**Person(s) responsible for administering treatment**

The daily supplements to pregnant women will be administered by field assistants, supported by village assistants. Infant supplements will be given to mothers on a weekly basis, but the mother/guardian will be responsible for daily administration. IPT drugs will be prescribed by the study clinician / study midwives but be self-administered by the women in the study.

1. **For questionnaires/interviews, who will be conducting these?**

Trained field assistants and study midwives.

**B5 Data management and Statistical analysis**

###### Who is responsible for the statistical design and analysis of the study?

Dr Sophie Moore & Dr Tony Fulford.

1. **Who will be primarily responsible for database design and data management?**

Dr Sophie Moore, Mr Jahid Hasan (Database Developer, MRC Keneba) & Dr Tony Fulford.

1. **Will data be double entered and verified?**

Yes, all data will be double entered and verified.

d) The MRC Laboratories IT/Data Management section supports Microsoft Access as its database package. If you are planning to use something else, please indicate which package and give a brief rationale:

We will use Microsoft Access.

**B6 Expected outputs and Dissemination of results**

**(a) What are the expected outputs (publications) from this project?**

It is anticipated that the data will be published in at least one peer-reviewed journal. Components of the data may also be published as abstracts/conference proceedings where relevant.

**(b) What other arrangements will there be to disseminate the findings?**

Data will be presented within the MRC through internal seminars and annual reports. Data will be presented nationally within The Gambia at the regular NaNA/MRC Nutrition Programme meetings. The results will also be communicated with the RHT. NaNA will be responsible for disseminating key study findings to other government partners at their discretion. It is also hoped that the data will be presented at relevant international meetings.

1. **Ethical issues**

This section is particularly important to the Ethics Committee. Please consult the guidance notes for preparation information sheets and consent forms; the checklist for subject information sheets; the template consent form; the guidelines for scientists (EC); the guidelines for the Gambian initiative for DNA Collections, as appropriate.

**(a) Outline how the study will contribute to improving the health of people of The Gambia**

Deficiencies of both macro- and micronutrients are widespread across much of sub-Saharan Africa, including The Gambia. Such deficiencies have been linked to immediate immunodeficiencies and, more recently, to impaired infant immune development and subsequent longer-term functional immunity. Targeted supplementation with single nutrients has had some success in improving morbidity and mortality from common infections in infancy, but little is known about the impact and mechanisms of both pre- and post-natal nutritional supplementation on infant immune development.

The proposed study will investigate, through a randomised control design, the impact of combined pre- and post-natal nutritional supplementation on immune development in Gambian infants. The design of this study will help establish the most efficacious form of nutritional therapy for optimal immune development. Availability of such data will help agencies such as the National Nutrition Agency implement policy aimed at optimising nutritional status and immune function at a critical stage of development.

**(b) Summarise the potential risks and benefits to individuals, communities or country**

During the study period, individual women will have access to improved antenatal care, with early detection of pregnancy, frequent clinical review and obstetric ultrasound monitoring; such improved care will help with the early diagnosis of maternal anaemia, pre-eclampsia and pregnancy associated malaria, all of which are significant causes of maternal morbidity among rural Gambian women [29]. Transport to referral facilities will be provided for women likely to benefit from a hospital delivery. Shortly after delivery, women and their infants will be visited by an MRC midwife/nurse, and those requiring medical attention will be transferred to the clinic in Keneba. Mothers and their infants will then be seen on a regular basis by MRC personnel. This will enable the early detection and treatment of infant morbidities.

There are risks that the ingestion of high doses of specific micronutrients (especially iron and / or vitamin A) during pregnancy might be harmful, particularly in a malarious setting. The pre-natal micronutrient combination used in the current study protocol has already been used safely and with good patient compliance in previous pregnancy trials elsewhere [30]. A recent study in Ghana demonstrated a beneficial effect of the same weaning food fortificant, given to infants from 6-12 months of age, on infant growth and motor development, with no adverse effects reported [25]. It is therefore believed that the interventions in this study pose no risk to non-pregnant women or their infants.

**(c) How will informed consent be obtained?**

A trained field worker will explain the full details of the study to the subject, covering all aspects of the study as laid out in the Information Sheet (see Appendix IV). Illiterate subjects will additionally have the full Information Sheet read to them; literate subjects will be allowed to read the Information Sheet in their own time. Any questions that arise will be answered by the field personnel, or referred to the principal investigator for clarification. Subjects will also be given the possibility to speak to one of the study investigators (PI, study midwife, study clinician) if they wish. If the subject agrees to participate, written consent will be obtained (see Appendix V).

**(d) How will you ensure confidentiality of the data gathered?**

Once enrolled, subjects will be allocated an individual study identification number (ID). Following delivery, infants will be allocated their own ID number. These unique ID numbers will be used on all samples and data forms generated during the course of the study. Linkage of the ID back to the study subject will not be possible without a lookup table, which will only be held by key study personnel during the course of the study. Once data collection is complete, analysis will be performed on an anonymised copy of the data. At all stages, staff/collaborators responsible for sample analysis will be blinded as to the subject’s identification. Together, these processes will ensure complete confidentiality of the data gathered.

**(e) Is a consent form attached?**

Yes, see Appendix V.

**(f) Is a subject information sheet attached?**

Yes, see Appendix IV.

**(g) Is the questionnaire (if applicable) attached?**

Not applicable.

#### D Resources Requested

# D1 Summary and cost

The Committees need to be reassured that the resource implications of the study have been fully considered and that the resources are available or are being sought to complete the study. Please refer to the following guidance in completing the table overpage.

For all cost categories please indicate whether (i)internal funds are requested, (ii) whether you plan to vire uncommitted funds in an existing budget (if so please give budget code and title), or (iii) an external source. If the project has not already been fully costed for a funding application, please discuss the resources needed with the External Grants Coordinator, Finance Manager and the Director of Operations.

As a brief guide:

**Staff**  - please indicate which staff members will be working on the project and the percentage of their time they will commit. If new staff are required please indicate the grade at which the appointment will be made and whether recruitment is internal(existing staff currently on another project) local(The Gambia) subregional(West Africa) or international. For new staff to be employed by MRC, full staff costs (including social security contributions, recruitment etc) may be obtained from the Personnel Manager, or from a spreadsheet operated by the External Grants Coordinator.

**Consumables** – these include all laboratory consumables, medicines and other clinical supplies, questionnaire production, computer consumables, specialist stationery and other supplies particular to the project. Freight costs should be included. The Purchasing Department and Laboratory Manager will advise on costs.

**Access to existing equipment-** Please specify e.g number of computers, and for example access to vehicles

**Capital and minor equipment** – any equipment that needs to be bought, replaced or repaired for the project. Freight and installation costs should be included. For projects at the MRC Laboratories, the Laboratory Manager will advise about the availability of laboratory equipment.

**Laboratory services-** MRC laboratories has facilities for HLA typing, clinical microbiology (including TB) and routine haematology and biochemistry. Please indicate the number of samples to be processed in each area

**Transport** – if MRC transport is requested please indicate approximately how many kilometres of travel will be required. The Transport Manager will advise on the most cost-effective way of meeting the need given the resources available (vehicles, drivers etc). You should allow for local public transport costs for staff or study subjects , and night allowances for staff.

###### Space - indicate the requests for office space, freezer space(including liquid nitrogen storage) and laboratory space implied by the project and how it is suggested that these are met in discussion with the Scientific Administrator

**Conference visits and Presentations-** The Unit is particularly keen to support presentations by higher degree students and scientific officers. Please make the case here

**Other** – this may include, particularly for externally-funded projects, accommodation costs, clinical fees and other overhead charges, communication costs, training, meeting costs, library. Please discuss the availability of residential accommodation with the Housing Manager (short stays) or Director of Operations (longer term) and office space with the Scientific Administrator.

**D2 Resource Request Spreadsheet(available in Excel on request)**

**TIMELINE**

**Please indicate the period of activity of the project from 31.03.2003 –31.03.2004**

**NEW STAFF**

| **Grade/Band** | **Where recruited from** | **% time on project** | **Source of funding** |
| --- | --- | --- | --- |
| N/A |  |  |  |
|  |  |  |  |

**EXISTING STAFF**

| **Who(names needed)** | **Grade** | **% time** | **Source of funding** |
| --- | --- | --- | --- |
| Dr Sophie Moore | Band 3 | 50 | Nutrition QQ |
| Dr Momodou Darboe | D3 | 50 | Nutrition QQ |
| Mrs Fatou Sosseh | D1 | 75 | Nutrition QQ |
| Dr Patrick Nshe | D3 | 50 | Nutrition QQ |
| Dr Pa Tamba N’Gom | E1 | 10 | Nutrition QQ |
| Field work supervisor | B3 | 100 | Nutrition QQ |
| 2 x senior field assistants | B2 | 100 | Nutrition QQ |
| 12 x field assistants | B1 | 100 | Nutrition QQ |
| 2 x trekking nurses | B3 | 50 | Nutrition QQ |
| 2 x laboratory technician | B2 | 100 | Nutrition QQ |
|  |  |  |  |

**OFFICE, LAB, FREEZER SPACE**

| **What** | **Where** | **% use** |
| --- | --- | --- |
| Research room | Bakary Dibba Clinical Research Centre | Daily |
| Laboratory | Whitehead Building | Daily |
| -80 Freezer | Keneba Utility Room | 2 shelves |
|  |  |  |

**CAPITAL EQUIPMENT NEEDED >3000 pounds**

| **List:** | **Cost** | **Source of funds** |
| --- | --- | --- |
| Siemens P10-4 Transducer | £4,000 | Nutrition QQ |
|  |  |  |

**EXISTING EQUIPMENT TO WHICH ACCESS IS NEEDED**

| **What** | **How often** |
| --- | --- |
| Centrifuge | Daily |
| Flow cytometer* | Weekly |
| ELISA plate reader* | Weekly |
| Luminex* | Weekly |
|  |  |

* Infant arm only, so not throughout study protocol.

**NEW MINOR EQUIPMENT REQUIRED**

| **List:** | **Cost** | **Source of funds** |
| --- | --- | --- |
| Anthropometric equipment | £5,000 | Nutrition QQ |
|  |  |  |

**CONSUMABLES**

|  | **What** | **Cost** | **Source of funds** |
| --- | --- | --- | --- |
| **LAB** | Plasticware | £4,000 | Nutrition QQ |
|  | Laboratory assays | TBC* | Nutrition QQ |
|  |  |  |  |
| **DRUGS** | Nutritional supplements | £50,000 | Nutrition QQ |
|  |  |  |  |
| **STATIONERY** | Forms, printer cartridges, lab books etc. | £2,000 | Nutrition QQ |
|  |  |  |  |
| **COMMUNICATIONS** | Phone cards, fax, email | £3,000 | Nutrition QQ |
|  |  |  |  |

* Our intention is to establish all antibody assays in Fajara, in collaboration with the infant immunology group. We are therefore unable to estimate costs at this stage.

**TRANSPORT AND LOCAL TRAVEL**

| **What:** | **Km** | **Cost** | **Source** |
| --- | --- | --- | --- |
| **Land Rover usage** | 2 vehicles* |  | Nutrition QQ |
| **Motorcycle usage** | 12 motorbikes* |  | Nutrition QQ |
| **Allowances** |  |  |  |
| **Fares** |  |  |  |

* To be allocated from Keneba pool, and charged centrally.

**USE OF LABORATORY SERVICES**

| **What:** | **No of specimens** | **Cost** | **Source** |
| --- | --- | --- | --- |
| **HLA Typing** | N/A |  |  |
| **Clinical Microbiology** | N/A |  |  |
| **Haematology/**  **Biochemistry** | CellDyn (Keneba) | £5,000 | Nutrition QQ |
| **Serology** | N/A |  |  |
| **HIV testing** | N/A |  |  |

**CONFERENCE PARTICIPATION**

| **Who/Where** | **When** | **Cost** | **Source** |
| --- | --- | --- | --- |
| N/A |  |  |  |
|  |  |  |  |
|  |  |  |  |

**D3 Sources of funds**

If external funds have been or will be sought, please state the progress of the application(s).

**Appendix I**

**Study timeline**

|  | 2008 | | | 2009 | | | | 2010 | | | | 2011 | | | | 2012 |
| --- | --- | --- | --- | --- | --- | --- | --- | --- | --- | --- | --- | --- | --- | --- | --- | --- |
|  | A-J | J-S | O-D | J-M | A-J | J-S | O-D | J-M | A-J | J-S | O-D | J-M | A-J | J-S | O-D | J-M |
| Phase I |  |  |  |  |  |  |  |  |  |  |  |  |  |  |  |  |
| Phase II |  |  |  |  |  |  |  |  |  |  |  |  |  |  |  |  |
| Phase III |  |  |  |  |  |  |  |  |  |  |  |  |  |  |  |  |
| Phase IV |  |  |  |  |  |  |  |  |  |  |  |  |  |  |  |  |
| Phase V |  |  |  |  |  |  |  |  |  |  |  |  |  |  |  |  |
|  |  |  |  |  |  |  |  |  |  |  |  |  |  |  |  |  |

**Phase I**: Preparation of SCC/EC documentation; liaison with NaNA & other collaborators; study protocol development; staff training; village meetings; registration of all women of reproductive age

**Phase II**: Recruitment of pregnant women at 12 wks gestation; First phase randomisation

**Phase III**: Deliveries

**Phase IV**: 6 month infant visits; Second phase randomisation

**Phase V**: 12 month infant visits

**Phase VI**: (not illustrated, April – September 2012) 6 months data analysis and write up

**Appendix II**

**Village listing for inclusion in the current application including the number of births (2007) and number of women aged 18-45 years (May 2008) registered within the West Kiang Demographic Surveillance System.**

| Village No | Village Name | No of Births, 2007 | Women 18-45 y, May 2008 |
| --- | --- | --- | --- |
| 001 | Dumbuto | 27 | 113 |
| 002 | Sankandi | 22 | 94 |
| 003 | Nyorro Jataba | 48 | 190 |
| 004 | Jatabba | 29 | 136 |
| 005 | Jiffarong | 56 | 213 |
| 006 | Bajana | 24 | 81 |
| 007 | Kuli Kunda | 24 | 121 |
| 008 | Jamaru | 4 | 14 |
| 009 | Brikamanding | 4 | 12 |
| 010 | Kantong Kunda | 24 | 86 |
| 011 | Jali | 40 | 183 |
| 013 | Manduar | 20 | 86 |
| 014 | Bang Kuling | 0 | 5 |
| 015 | Gissay | 8 | 17 |
| 016 | Tankular | 24 | 124 |
| 017 | Joli | 16 | 75 |
| 018 | Kuyang | 4 | 15 |
| 019 | Bantasu | 1 | 1 |
| 020 | Santamba | 0 | 1 |
| 021 | Missira | 5 | 10 |
| 022 | Taborangkoto | 5 | 22 |
| 023 | Burong | 14 | 68 |
| 024 | Jula Kunda | 11 | 45 |
| 025 | Karantaba | 27 | 130 |
| 026 | Mandina | 6 | 28 |
| 027 | Janneh Kunda | 30 | 113 |
| 028 | Kemoto | 22 | 57 |
| 029 | Keneba | 69 | 292 |
| 030 | Batelling | 8 | 38 |
| 031 | Sandeng | 5 | 38 |
| 032 | Wudeba | 3 | 15 |
| 033 | Sinchu Njanko | 1 | 5 |
| 034 | Kenokoto | 1 | 7 |
| 035 | Manari | 1 | 2 |
| 036 | Nineteen | 0 | 3 |
| **Totals** |  | **583** | **2440** |

# Appendix III

# Sample collection schedule

|  | Pre-natal (gestational age) | | | Delivery | Post-natal (infant age) | | | | |
| --- | --- | --- | --- | --- | --- | --- | --- | --- | --- |
|  | 12 wk | 20 wk | 30 wk |  | 1 wk | 8 wk | 12 wk | 24 wk | 52 wk |
| **Mother** |  |  |  |  |  |  |  |  |  |
| Venous blood | X | X | X |  |  |  |  |  |  |
| Breast milk |  |  |  |  |  | X | X | X | X |
|  |  |  |  |  |  |  |  |  |  |
| **Delivery** |  |  |  |  |  |  |  |  |  |
| Cord blood |  |  |  | X |  |  |  |  |  |
| Placenta sample |  |  |  | X |  |  |  |  |  |
|  |  |  |  |  |  |  |  |  |  |
| **Infant** |  |  |  |  |  |  |  |  |  |
| Thymic US |  |  |  |  | X | X |  | X | X |
| Venous blood |  |  |  |  |  |  | X | X | X |
|  |  |  |  |  |  |  |  |  |  |

**Sample analysis details:**

Mother

Venous bleed (10mL) at 12, 20 & 30 wks gestation for assessment of plasma micronutrient status and standard haematology.

Breast milk sample collection (5mL from each breast) at 1, 8, 12, 24 & 52 weeks post-partum for assessment of breast milk micronutrient status.

Delivery

Cord blood (10mL) for the analysis of micronutrient status and immune parameters.

Placental biopsy: 2 x 2 cm^3^ full-thickness biopsy for malaria histology.

Infant

Venous bleed (3mL at 12 wk, 5mL at 24 & 52 wk of age) for assessment of antibody response to vaccination and lymphocyte subset phenotyping, enumeration and function (24 & 52 wk only).

# Appendix IV

# Medical Research Council Laboratories

**A randomized trial to investigate the effects of pre-natal and infancy nutritional supplementation on infant immune development**

**(SCC 1126)**

**Subject Information Sheet**

Version number 02 18^th^ July, 2008

(If necessary, to be read to participants in their own language)

**What is the purpose of this study?**

The diet of many women in West Kiang is often poor. This sometimes leads to problems during pregnancy, such as anaemia, and may also slow the growth and development of babies. In addition, the types of foods currently fed to infants beyond the period when exclusive breast feeding is no longer adequate may be lacking in important nutrients and this may cause poor growth and increased susceptibility to infections. We believe that providing additional nutrients to women during pregnancy and then to their infants from 6 months of age will help improve the growth and health of infants. We would like to look at this in more detail in West Kiang and would like to invite you to participate.

Before you decide to participate it is important for you to understand why the research is being done and what it will involve. Please take time to listen to this carefully and discuss it with others if you wish. Please ask if there is anything which is unclear or if you would like more information. Take time to decide whether or not you wish to take part.

**What does participation in the study mean for you?**

We are trying to find out if improving your nutrition during pregnancy can help improve your infant’s immune system – that is to improve the body systems that help them to fight infection. We also want to find out if improving the nutrient content of weaning foods given to infants can help improve their immune system. To do this, we would like to enrol you in this study and if you become pregnant, we would like to conduct a series of tests and measures on you and then on your infant when he/she is born.

The following will be required if you agree to take part:

Once enrolled, you will be visited every month by a member of the project team with a short questionnaire on the date of your last period. When you report missing 2 consecutive periods we will ask you to provide a small urine sample which we will use to test for pregnancy. You will then be visited by the study midwife to confirm the result of this test and, if you are pregnant, you will be invited to come to MRC Keneba for an initial study visit. If you are not pregnant, you will continue to be visited each month by the field worker.

At this visit in Keneba, you will have a routine antenatal check by an MRC midwife. During this time, we would also like to measure the size of your developing baby using a process known as ultrasound. This process is painless and harmless to both you and your baby. We will also measure your height, weight, waist and hip circumference, and also your body composition (how much fat you have in your body). All of these processes are painless. Finally, we would like to collect a small blood sample from one of your veins so that we can measure the amount of certain nutrients in your blood. The amount we will collect is very small (10mls in total, equivalent to 2 teaspoons). You will also be invited back to Keneba when you are 20 and 30 weeks pregnant for exactly the same measurements to be made.

After this first visit, you will be randomized to receive one of 4 different types of supplement. These will be in the form of either tablets or a food-based supplement and you will be asked to take these supplements everyday until the delivery of your child. All of these supplements contain iron and folate, as is normally given to pregnant women in West Kiang, so will replace the tablets you would normally receive.

When you are in labour, an MRC field assistant living in or close to your village will be informed. Following delivery, and if we have your permission, he/she will collect your placenta from the midwife/traditional birth attendant and will use this to take samples of cord blood and placental material. These samples will then be transported back to the laboratory in Keneba for storage and later used to measure markers of nutritional status and infection.

At 1, 8, 12 and 24 weeks of age, we will invite you and your infant to Keneba for some further measurements. Firstly, we will use the same ultrasound we used on you during pregnancy to assess the size of an organ in your infant’s chest known as the thymus. These measurements are painless and harmless to your infant and will only take a few minutes. We will also weigh and measure your child and take a small blood sample from one of their veins so that we can measure certain markers of immune function, such as how well your infant has responded to vaccination. The amount we will collect is very small (3-5mls in total, equivalent to 1 teaspoon) and will be used to measure a number of different markers of immune function in your child. In addition we also wish to use these blood samples, and the samples collected from you during pregnancy, to extract DNA so that we can look for genes such as those related to growth and immune development. We will also ask you to provide a small amount of breast milk so that we can measure the micronutrient content of your milk. From when your child is six months of age, we will ask you to add a supplement to their normal weaning food. Two supplements are included, one that contains extra nutrients and one that doesn’t. We will ask you to add this supplement to your infant’s food on a daily basis until the infant reaches 12 months of age. Your participation in the study will end when your infant reaches his/her first birthday.

At the end of the study, some of the blood samples collected will be transferred to a laboratory in the UK for analysis. This is because we do not have the equipment in The Gambia for measuring some of the factors we are interested in, such as nutrient levels in your blood. All of the information obtained from the UK will then be sent back to the investigators in The Gambia.

All information which is collected during the course of this study will be kept strictly confidential and you will only be identified by an ID number.

It is up to you to decide whether or not to take part. If you do decide to take part you will be asked to sign a consent form. If you decide to take part you are still free to withdraw at any time and without giving a reason. If you have any questions, please ask. We hope that you will agree to participate.

Thank you very much.

**Principal investigator:** Dr Sophie Moore

Head of Station, MRC Keneba.

Mobile number: 9963991

# Appendix V

# Medical Research Council Laboratories

**A randomized trial to investigate the effects of pre-natal and infancy nutritional supplementation on infant immune development**

**(SCC 1126)**

**Consent Form:**

Version number 02 18^th^ July, 2008

The information sheet has been read to me and I understand it / I have read and understood the information sheet.

I understand what participation in the study means for me and my infant.

I understand that the information regarding me that is collected in the course of this study will remain confidential.

I understand that from early in pregnancy until delivery I will be asked to take a daily nutritional supplement. I also understand that from when my child is 6 months of age, I will be asked to give him/her an additional food supplement or placebo everyday and until he/she reaches 12 months.

I understand that laboratory tests will be done on the blood, breast milk and placental samples from me and my infant, and that some of these samples may later be sent to the UK for laboratory testing. I also understand that part of the blood samples collected will be stored for future genetic analyses conducted by the investigators running this study.

I understand that if either myself or my infant gets sick during the study period, I can go to the clinic where study staff are providing care, and that we will be examined and treated for free.

I understand that I am free to take part in the study or refuse, and that I can withdraw either myself of my infant from the study at any time, and without giving any reason. Deciding not to take part or to withdraw from the study will not affect the care that I or any of my family are normally entitled to.

I have had a chance to ask questions and have them answered.

Signature or thumb print of volunteer: _ _ _ _ _ _ _ _ _ _ _ _ _ _ _ _ _

This form has been read by / I have read the above to _ _ _ _ _ _ _ _ _ _ _ _ _ _

(write name of volunteer)

in a language that she understands. I believe that she has understood what I explained and that she has freely agreed to take part in the study.

Signature of field worker: _ _ _ _ _ _ _ _ _ _ _ _ _ _ _ _ _ _ _ _ _

Name of field worker: _ _ _ _ _ _ _ _ _ _ _ _ _ _ _ _ _ _ _ _ _

Date: |___|___| / |___|___| / |___|___|___|___|
