# Supplementary figures and images for: Impact of nutritional supplementation during pregnancy on antibody responses to diphtheria-tetanus-pertussis vaccination in infants: A randomised trial in The Gambia
Source: PLoS Med. 2019 Aug 6;16(8):e1002854. doi: 10.1371/journal.pmed.1002854 (PMC6684039; doi:10.1371/journal.pmed.1002854)

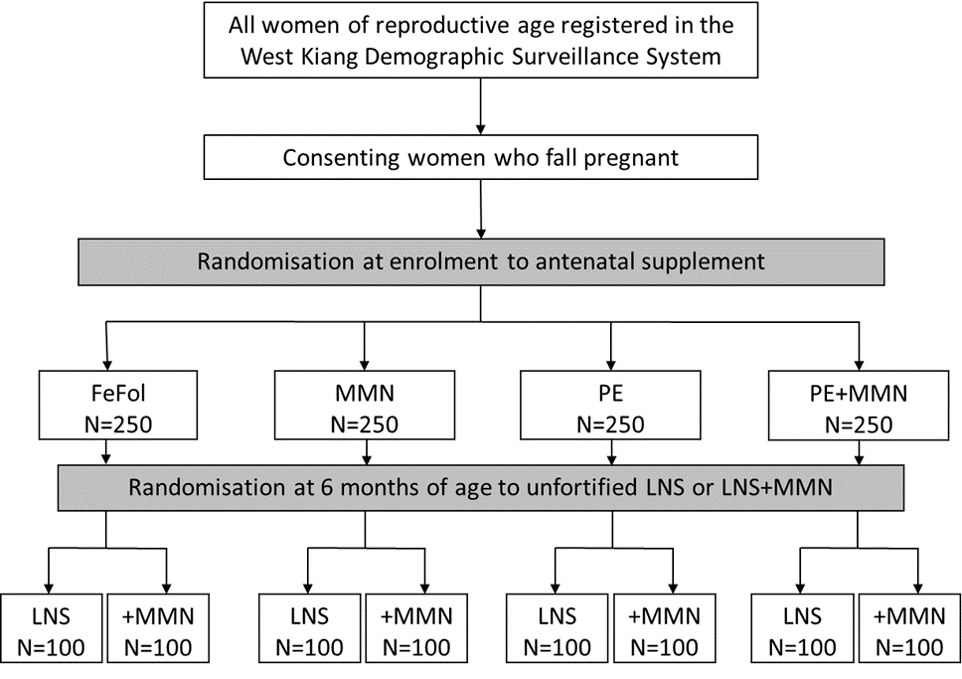

Supplement: S1 Fig — FeFol, iron folic acid; LNS, lipid-based nutritional supplement; MMN, multiple micronutrients; PE, protein-energy. (TIF) [file pmed.1002854.s005.tif]
