# Supplementary material for: Impact of nutritional supplementation during pregnancy on antibody responses to diphtheria-tetanus-pertussis vaccination in infants: A randomised trial in The Gambia
Source: PLoS Med. 2019 Aug 6;16(8):e1002854. doi: 10.1371/journal.pmed.1002854 (PMC6684039; doi:10.1371/journal.pmed.1002854)
Supplement: S1 Table — (DOCX) [file pmed.1002854.s006.docx]

**S1 Table. Comparisons of characteristics between infants with antibody measurements at 12 weeks** **of age and those missing data^a^**

| **Maternal variables** | **Antibody data at 12 weeks (*n*=710)** | **Missing 12 weeks antibody data (*n*=90)** | ***p-value^f^*** |
| --- | --- | --- | --- |
| **Enrolment** |  |  |  |
| Age at enrolment (years) | 29.8 (6.4) | 30.2 (6.8) | 0.332 |
| Parity, *n* | 4 .0 (2.6) | 4.2 (2.7) | 0.475 |
| Formal education, *n* (%) | 152 (21.8) | 16 (21.1) | 0.309 |
| Gestational age at enrolment (weeks) | 13.6 (3.1) | 13.7 (3.3) | 0.383 |
| BMI at enrolment (kg/m^2^) | 20.9 (3.2) | 21.2 (3.5) | 0.474 |
| Hb at enrolment (g/dL) | 11.2 (1.4) | 11.4 (1.4) | 0.096 |
| Anaemia at enrolment, *n* (%)^b^ | 238 (37.7) | 52 (42.6) | 0.302 |
| **30 weeks** |  |  |  |
| Hb at 30 weeks gestation (g/dL) | 10.4 (1.5) | 10.7 (1.3) | 0.067 |
| Anaemia at, 30 weeks, *n* (%)^b^ | 360 (58.7) | 61 (51.3) | 0.132 |
| **Throughout pregnancy** |  |  |  |
| Morbidity events^c^ | 4.5 (4.8) | 5.2 (6.6) | 0.229 |
| Compliance to supplement^d^ (% (SD)) | 90.6 (9.9) | 88.4 (10.0) | 0.769 |
| **Infant variables** |  |  |  |
| **Birth** |  |  |  |
| Gestational age at delivery (weeks) | 40.2 (1.9) | 40.2 (1.4) | 0.218 |
| Sex, *n* (%): male | 366 (51.6) | 36 (46.2) | 0.366 |
| Dry season birth^e^, *n* (%): | 443 (62.4) | 45 (57.7) | 0.417 |
| Birth weight (kg) | 3.06 (0.40) | 3.01 (0.40) | 0.348 |
| Birth length (cm) | 49.57 (1.9) | 49.68 (1.9) | 0.504 |

^a^Values are means (SD) unless stated otherwise

^b^Anemia was defined as a haemoglobin level between 7.0 and 10.9 g/dL (WHO).

^c^Number of morbidity episodes between enrolment and delivery

^d^Compliance percentage was generated by dividing the number of LNS jars or tablets the women consumed by the number she received, and multiplying by 100

^e^Dry season = November to May

^f^P-values were calculated by Student’s t-test with Welch’s correction for unequal sample sizes.
