# Supplementary material for: Impact of nutritional supplementation during pregnancy on antibody responses to diphtheria-tetanus-pertussis vaccination in infants: A randomised trial in The Gambia
Source: PLoS Med. 2019 Aug 6;16(8):e1002854. doi: 10.1371/journal.pmed.1002854 (PMC6684039; doi:10.1371/journal.pmed.1002854)
Supplement: S2 Table — (DOCX) [file pmed.1002854.s007.docx]

**S2 Table. Comparisons of characteristics between infants with antibody measurements at 24 weeks of age and those missing data^a^**

| **Maternal variables** | **Antibody data at 24 weeks (*n*=662)** | **Missing 24 weeks antibody data (*n*=138)** | **p-value^f^** |
| --- | --- | --- | --- |
| **Enrolment** |  |  |  |
| Age at enrolment (years) | 29.6 (6.7) | 30.3 (6.8) | 0.641 |
| Parity | 4.0 (2.8) | 4.2 (2.7) | 0.475 |
| Formal education, *n* (%) | 137 (21.1) | 31 (25.2) | 0.309 |
| Gestational age at enrolment (weeks) | 13.9 (3.2) | 13.7 (3.3) | 0.619 |
| BMI at enrolment (kg/m^2^) | 20.9 (3.2) | 21.2 (3.5) | 0.474 |
| Hb at enrolment (g/dL) | 11.2 (1.3) | 11.4 (1.4) | 0.278 |
| Anaemia at enrolment, *n* (%)^b^ | 32 (43.2) | 258 (36.3) | 0.242 |
| **30 weeks** |  |  |  |
| Hb at 30 weeks gestation (g/dL) | 10.7 (1.4) | 10.7 (1.3) | 0.067 |
| Anaemia at, 30 weeks, *n* (%)^b^ | 50 (67.6) | 371 (56.4) | 0.065 |
| **Throughout pregnancy** |  |  |  |
| Morbidity events^c^ | 5.3 (5.9) | 5.1 (6.6) | 0.229 |
| Compliance to supplement^d^ (% (SD)) | 88.0 (15.3) | 88.7 (13.7) | 0.769 |
| **Infant variables** |  |  |  |
| **Birth** |  |  |  |
| Gestational age at delivery (weeks) | 40.3 (1.6) | 40.2 (1.4) | 0.218 |
| Sex, *n* (%): male | 337 (50.9) | 65 (51.6) | 0.889 |
| Dry season birth^e^, *n* (%): | 259 (39.1) | 41 (32.5) | 0.163 |
| Birth weight (kg) | 3.04 (0.41) | 3.01 (0.40) | 0.348 |
| Birth length (cm) | 49.55 (1.82) | 49.62 (2.0) | 0.725 |

^a^Values are means (SD) unless stated otherwise

^b^Anemia was defined as a haemoglobin level between 7.0 and 10.9 g/dL (WHO).

^c^Number of morbidity episodes between enrolment and delivery

^d^Compliance percentage was generated by dividing the number of LNS jars or tablets the women consumed by the number she received, and multiplying by 100

^e^Dry season = November to May

^f^P-values were calculated by Student’s t-test with Welch’s correction for unequal sample sizes.
