# Supplementary material for: Impact of nutritional supplementation during pregnancy on antibody responses to diphtheria-tetanus-pertussis vaccination in infants: A randomised trial in The Gambia
Source: PLoS Med. 2019 Aug 6;16(8):e1002854. doi: 10.1371/journal.pmed.1002854 (PMC6684039; doi:10.1371/journal.pmed.1002854)
Supplement: S3 Table — DTP, diphtheria-tetanus-pertussis. (DOCX) [file pmed.1002854.s008.docx]

**S3 Table. Infants responder *n* (%) to each DTP vaccine antigens by supplement groups^a^**

|  | **Maternal supplement groups** | | | |  |
| --- | --- | --- | --- | --- | --- |
|  | **Tablet supplements** | | **LNS** | |  |
| **Vaccine antigen** | **FeFol**  **(*n*=178)** | **MMN**  **(*n*=179)** | **PE**  **(*n*=170)** | **PE+MMN**  **(*n*=182)** | ***p-value^b^*** |
| **12 weeks** |  |  |  |  |  |
| Diphtheria | 84 (47.2) | 75 (41.9) | 77 (45.3) | 80 (43.7) | 0.777 |
| Tetanus | 173 (97.2) | 174 (97.2) | 166 (97.7) | 178 (97.3) | 0.993 |
| Pertussis | 83 (46.6) | 89 (49.7) | 97 (57.1) | 85 (46.5) | 0.163 |
| **24 weeks** |  |  |  |  |  |
| Diphtheria | 162 (97) | 156 (95.7) | 158 (97.5) | 165 (97.1) | 0.807 |
| Tetanus | 167 (100) | 162 (99.4) | 161 (99.4) | 169 (99.4) | 0.797 |
| Pertussis | 149 (89.2) | 134 (82.2) | 146 (90.1) | 155 (91.2) | 0.050 |

^a^Being responder to diphtheria or tetanus vaccines was defined as presenting an antibody titre >0.1 IU/ml according to international standards (WHO) and for pertussis as an in-house antibody assay was used an arbitrary threshold was established at >5.0 EU/ml.

^b^P-values were obtained from comparisons across the supplement groups.
