# Supplementary material for: Impact of nutritional supplementation during pregnancy on antibody responses to diphtheria-tetanus-pertussis vaccination in infants: A randomised trial in The Gambia
Source: PLoS Med. 2019 Aug 6;16(8):e1002854. doi: 10.1371/journal.pmed.1002854 (PMC6684039; doi:10.1371/journal.pmed.1002854)
Supplement: S4 Table — CI, confidence interval; DTP, diphtheria-tetanus-pertussis. (DOCX) [file pmed.1002854.s009.docx]

**S4 Table. Comparisons of the unadjusted means (95% confidence intervals) of diphtheria, tetanus and pertussis antibody titres at 12 weeks of age, following the first DTP vaccination, by supplement groups**

|  |  | | **Effect size (95%CI) (%) of the comparisons between the supplement groups^b^** | | | | | |
| --- | --- | --- | --- | --- | --- | --- | --- | --- |
| **Vaccine antigen** | | **Means^a^ (95% CI)** | **FeFol** | ***p-value^c^*** | **MMN** | ***p-value^c^*** | **PE** | ***p-value^c^*** |
| **Diphtheria** |  | |  |  |  |  |  |  |
| FeFol | 0.11 (0.08, 0.15) | | Reference |  |  |  |  |  |
| MMN | 0.13 (0.10, 0.17) | | -6.6 (-23.4, 10.2) | 0.443 | Reference |  |  |  |
| PE | 0.12 (0.09, 0.16) | | -2.3 (-19.6, 15.0) | 0.798 | 4.3 (-12.7, 21.3) | 0.618 | Reference |  |
| PE+MMN | 0.14 (0.11, 0.19) | | -10.4 (-27.9, 7.1) | 0.244 | -3.8 (-21.1, 13.4) | 0.664 | -8.1 (-25.9, 9.6) | 0.368 |
| **Tetanus** |  | |  |  |  |  |  |  |
| FeFol | 0.58 (0.50, 0.66) | | Reference |  |  |  |  |  |
| MMN | 0.69 (0.60, 0.80) | | -7.9 (-16.6, 0.8) | 0.074 | Reference |  |  |  |
| PE | 0.66 (0.57, 0.76) | | -5.5 (-14.0, 3.0) | 0.205 | 2.4 (-6.5, 11.3) | 0.592 | Reference |  |
| PE+MMN | 0.62 (0.54, 0.72) | | -3.3 (-12.0, 5.4) | 0.456 | 4.6 (-4.4, 13.7) | 0.313 | 2.2 (-6.7, 11.1) | 0.625 |
| **Pertussis** |  | |  |  |  |  |  |  |
| FeFol | 5.99 (5.00, 7.18) | | Reference |  |  |  |  |  |
| MMN | 5.57 (4.65, 6.67) | | 3.2 (-7.9, 14.2) | 0.573 | Reference |  |  |  |
| PE | 4.61 (3.88, 5.48) | | 11.4 (0.50, 22.2) | **0.040** | 8.2 (-2.6, 19.1) | 0.136 | Reference |  |
| PE+MMN | 5.93 (4.93, 7.13) | | 0.50 (-10.7, 11.6) | 0.935 | -2.7 (-13.9, 8.5) | 0.634 | -10.9 (-21.9, 0.10) | 0.052 |

FeFol, iron-folic acid (reference); MMN, multiple micronutrient; PE, protein-energy, PE+MMN, protein energy combined with multiple micronutrients.

^a^Antibody concentrations were log-transformed and for reporting, mean values and confidence intervals were back-transformed from the logarithm scale and expressed in IU/ml for diphtheria and tetanus antibody titres and in EU/ml for pertussis antibody titres.

^b^Effect sizes were determined using the mean difference between two supplement groups from the Student’s t-test and were expressed as percentage (%).

^c^P-values were calculated by Student’s t-test on the log-transformed antibody concentrations.
