# Supplementary material for: Impact of nutritional supplementation during pregnancy on antibody responses to diphtheria-tetanus-pertussis vaccination in infants: A randomised trial in The Gambia
Source: PLoS Med. 2019 Aug 6;16(8):e1002854. doi: 10.1371/journal.pmed.1002854 (PMC6684039; doi:10.1371/journal.pmed.1002854)
Supplement: S5 Table — CI, confidence interval; DTP, diphtheria-tetanus-pertussis. (DOCX) [file pmed.1002854.s010.docx]

**S5 Table. Comparisons of the unadjusted means (95% confidence intervals) of diphtheria, tetanus and pertussis antibody titres at 24 weeks of age, following three DTP vaccination, by supplement groups**

|  |  | | **Effect size (95%CI) (%) of the comparisons between the supplement groups^b^** | | | | | |
| --- | --- | --- | --- | --- | --- | --- | --- | --- |
| **Vaccine antigen** | | **Means^a^ (95% CI)** | **FeFol** | ***p-value^c^*** | **MMN** | ***p-value^c^*** | **PE** | ***p-value^c^*** |
| **Diphtheria** |  | |  |  |  |  |  |  |
| FeFol | 1.35 (1.17, 1.57) | | Reference |  |  |  |  |  |
| MMN | 1.37 (1.17, 1.61) | | -0.6 (-10, 8.9) | 0.909 | Reference |  |  |  |
| PE | 1.43 (7.86, 1.65) | | -2.2 (-11.3, 6.8) | 0.628 | -1.7 (-11.2, 7.8) | 0.728 | Reference |  |
| PE+MMN | 1.46 (1.26, 1.70) | | -3.3 (-12.4, 5.8) | 0.476 | -2.7 (-12.3, 6.8) | 0.571 | -1.1 (-10.2, 8.1) | 0.818 |
| **Tetanus** |  | |  |  |  |  |  |  |
| FeFol | 3.61 (3.06, 4.26) | | Reference |  |  |  |  |  |
| MMN | 3.64 (3.04, 4.35) | | -0.4 (-10.9, 10.2) | 0.946 | Reference |  |  |  |
| PE | 3.73 (3.13, 4.45) | | -1.4 (-11.9, 9.1) | 0.788 | -1.1 (-12, 9.8) | 0.846 | Reference |  |
| PE+MMN | 4.01 (3.39, 4.75) | | -4.6 (-14.9, 5.7) | 0.379 | -4.2 (-14.9, 6.4) | 0.435 | -3.2 (-13.8, 7.4) | 0.558 |
| **Pertussis** |  | |  |  |  |  |  |  |
| FeFol | 80.04 (60.27, 106.31) | | Reference |  |  |  |  |  |
| MMN | 67.25 (48.72, 92.83) | | 7.6 (-11, 26.1) | 0.423 | Reference |  |  |  |
| PE | 101.07 (75.58, 135.16) | | -10.1 (-27.7, 7.4) | 0.258 | -17.7 (-36.5, 1.1) | 0.065 | Reference |  |
| PE+MMN | 118.71 (91.28, 154.39) | | -17.1 (-33.8, -0.4) | **0.045** | -24.7 (-42.6, -6.8) | **0.007** | -7 (-23.9, 9.9) | 0.417 |

FeFol, iron-folic acid (reference); MMN, multiple micronutrient; PE, protein-energy, PE+MMN, protein energy combined with multiple micronutrients.

^a^Antibody concentrations were log-transformed and for reporting, mean values and confidence intervals were back-transformed from the logarithm scale and expressed in IU/ml for diphtheria and tetanus antibody titres and in EU/ml for pertussis antibody titres.

^b^Effect sizes were determined using the mean difference between two supplement groups from the Student’s t-test and were expressed as percentage (%).

^c^P-values were calculated by Student’s t-test on the log-transformed antibody concentrations.
