# Supplementary material for: Impact of nutritional supplementation during pregnancy on antibody responses to diphtheria-tetanus-pertussis vaccination in infants: A randomised trial in The Gambia
Source: PLoS Med. 2019 Aug 6;16(8):e1002854. doi: 10.1371/journal.pmed.1002854 (PMC6684039; doi:10.1371/journal.pmed.1002854)
Supplement: S6 Table — CI, confidence interval; DTP, diphtheria-tetanus-pertussis. (DOCX) [file pmed.1002854.s011.docx]

**S6 Table. Comparisons of the ratios of the unadjusted means (95% confidence intervals) of diphtheria, tetanus and pertussis antibody titres between 12 and 24 weeks of age, following three DTP vaccinations, by supplement groups**

|  |  | | **Effect size (95%CI) (%) of the comparisons between the supplement groups^b^** | | | | | |
| --- | --- | --- | --- | --- | --- | --- | --- | --- |
| **Vaccine antigen** | | **Means^a^ (95% CI)** | **FeFol** | ***p-value^c^*** | **MMN** | ***p-value^c^*** | **PE** | ***p-value^c^*** |
| **Diphtheria** |  | |  |  |  |  |  |  |
| FeFol | 10.2 (7.4, 14.8) | | Reference |  |  |  |  |  |
| MMN | 10.9 (8.2, 14.9) | | 3.8 (-14.4, 22) | 0.685 | Reference |  |  |  |
| PE | 9.9 (7.6, 13.4) | | 0 (-18.3, 18.4) | 0.996 | -3.7 (-22, 14.6) | 0.690 | Reference |  |
| PE+MMN | 11.3 (8.5, 15.4) | | 8.1 (-10.8, 26.9) | 0.399 | 4.3 (-14.5, 23.2) | 0.651 | 8 (-10.9, 27) | 0.405 |
| **Tetanus** |  | |  |  |  |  |  |  |
| FeFol | 5.8 (4.7, 7.4) | | Reference |  |  |  |  |  |
| MMN | 5.1 (4.1, 6.4) | | 10.9 (-2.8, 24.6) | 0.119 | Reference |  |  |  |
| PE | 4.7 (3.8, 6.1) | | 6.2 (-7, 19.5) | 0.353 | -4.6 (-19.1, 9.8) | 0.528 | Reference |  |
| PE+MMN | 6.0 (4.7, 8.0) | | -1.6 (-15, 11.9) | 0.820 | -12.4 (-27.1, 2.2) | 0.096 | -7.8 (-22, 6.4) | 0.281 |
| **Pertussis** |  | |  |  |  |  |  |  |
| FeFol | 16.2 (12.0, 22.6) | | Reference |  |  |  |  |  |
| MMN | 16.8 (12.1, 24.2) | | 4.3 (-16.3, 24.9) | 0.683 | Reference |  |  |  |
| PE | 23.0 (16.5, 33.4) | | -18 (-38.1, 2.1) | 0.080 | -22.3 (-43.1, -1.4) | **0.036** | Reference |  |
| PE+MMN | 24.8 (18.6, 34.1) | | -16.2 (-36.5, 4.1) | 0.117 | -20.5 (-41.5, 0.5) | 0.055 | 1.8 (-18.7, 22.3) | 0.865 |

FeFol, iron-folic acid (reference); MMN, multiple micronutrient; PE, protein-energy, PE+MMN, protein energy combined with multiple micronutrients.

^a^Antibody concentrations were log-transformed and for reporting, mean values and confidence intervals were back-transformed from the logarithm scale and expressed in IU/ml for diphtheria and tetanus antibody titres and in EU/ml for pertussis antibody titres.

^b^Effect sizes were determined using the mean difference between two supplement groups from the Student’s t-test and were expressed as percentage (%).

^c^P-values were calculated by Student’s t-test on the log-transformed antibody concentrations.
