# Supplementary material for: Impact of nutritional supplementation during pregnancy on antibody responses to diphtheria-tetanus-pertussis vaccination in infants: A randomised trial in The Gambia
Source: PLoS Med. 2019 Aug 6;16(8):e1002854. doi: 10.1371/journal.pmed.1002854 (PMC6684039; doi:10.1371/journal.pmed.1002854)
Supplement: S4 Appendix — (PDF) [file pmed.1002854.s004.pdf]

The Gambia Government / MRC Laboratories Joint
**ETHICS COMMITTEE**

c/o MRC Laboratories Fajara
P. O. Box 273, Banjul
The Gambia, West Africa

Fax: +220 – 4494498 or 4496 513
Tel: +220 – 4494 073-9 ext. 2308

20th August 2008

Dr Sophie Moore
Head of Station
MRC Kenaba

Dear Dr. Moore

**Re: SCC 1126v2. 21st July 2008. A randomized trial to investigate the effects of pre-natal and infancy nutritional supplementation on infant immune development.**

Thank you for your response to the concerns raised by the Ethics Committee on your study to investigate the effects of prenatal and infancy nutritional supplementation on infant immune development.

We have noted your confirmation that as part of the Trial Monitoring/GCP requirements for such a trial, you will set up a Data and Safety Monitoring Committee for whom un-blinded results on outcomes will be made available. We are also pleased to note that you have already consulted Dr Jenny Mueller, Clinical Trials Support Manager, MRC Fajara, regarding the study design and that she is providing helpful input into the monitoring aspects required.

This proposal now has our Committee's full approval to proceed.

Best wishes

Yours sincerely

Mr. Malcolm Clarke
Chairman, Gambia Government/MRC Joint Ethics Committee

**The Gambia Government / MRC Laboratories Joint Ethics Committee:**

*Mr Malcolm Clarke, Chairman*
*Mrs Kathy Hill, Secretary*
*Mrs Norwoh Ali, 2nd Secretary*
*Professor Ousman Nyan, Scientific Advisor*
*Mr Dawda Jagne*
*Mrs Bertha Mboge*
*Mr. Modou Phall*

*Professor Tumani Corrah*
*Professor Hilton Whittle*
*Dr Stephen Howie*
*Dr Mariatou Jallow*
*Dr. Lamin Sidibeh*
*Mr. Malamin Sonko*
